# Arm‐Length‐Controlled CsPbBr_3_ Nanocrystals for Tunable Optical and Assembly Behavior

**DOI:** 10.1002/adma.202519211

**Published:** 2026-03-24

**Authors:** Irina Skvortsova, Sudipta Seth, Juliette Zito, Robin Girod, Bob Van Hout, Annick De Backer, Tom Stoops, Sergey Abakumov, Evgenii Vlasov, Tejmani Behera, Sandra Van Aert, Elke Debroye, Johan Hofkens, Sara Bals

**Affiliations:** ^1^ Electron Microscopy for Materials Science (EMAT) & NANOlight Center of Excellence University of Antwerp Antwerp Belgium; ^2^ Department of Chemistry KU Leuven Leuven Belgium; ^3^ Max Planck Institute for Polymer Research Mainz Germany

**Keywords:** assemblies, blinking, electron tomography, halide perovskites

## Abstract

Colloidal cesium lead bromide (CsPbBr_3_) nanocrystals (NCs) are excellent candidates for various photonic and optoelectronic applications due to their bright and stable green emission. Here, we establish arm length control as a central structural parameter that governs both the optical properties of individual CsPbBr_3_ NCs and their self‐assembly behavior. Armed NCs, featuring a cubic core with multiple protruding arms, are synthesized by controlling seed size, concentration, and injection temperature, while arm length is tuned via cesium oleate concentration. Prolonged storage in toluene is shown to lead to a time‐dependent morphological evolution from armed NCs to 26‐faceted rhombicuboctahedra, with short‐armed structures as intermediates. NCs with a longer arm length yield enhanced radiative efficiency, extended photoluminescence (PL) lifetimes, and suppressed blinking, making such NCs suitable for light‐emitting devices and quantum photonic applications. In contrast, short‐armed NCs exhibit faster recombination, stronger PL intermittency, and increased surface accessibility, which are favorable for sensing and high‐speed single‐photon emission. The arm length also governs self‐assembly behavior, hereby opening new possibilities for applications. Armed NCs form densely 3D‐packed assemblies with tunable configurations. This work demonstrates how arm length tuning expands the functional potential of CsPbBr_3_ NCs by linking morphological control to both optical response and self‐assembly characteristics.

## Graphical abstract

In this work, the synthesis parameters that dictate the formation of armed CsPbBr_3_ nanocrystals are described, and routes enabling precise control over arm length are proposed. Direct links between morphology and optoelectronic behavior are established. Arm length is also shown to govern the geometry and packing efficiency of assembled superlattices, enabling new opportunities for functional CsPbBr_3_ nanocrystal‐based materials.

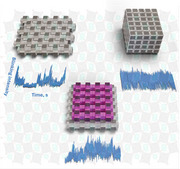

## 1 Introduction

Cesium lead bromide (CsPbBr_3_) nanocrystals (NCs) have emerged as highly promising materials for photonic and optoelectronic applications due to their exceptional optical properties, particularly their stable and efficient green emission [1, 2, 3, 4]. So far, the field has been predominantly focused on CsPbBr_3_ nanocubes [5, 6, 7, 8], which have long been considered to represent the most thermodynamically stable morphology. However, recent advances in colloidal synthesis have started to reveal a more diverse morphological landscape [9], introducing novel shapes such as 26‐faceted rhombicuboctahedra and 12‐faceted dodecahedra [10, 11], as well as so‐called armed structures [12], which refer to a class of colloidal perovskite NCs characterized by a central core, typically resembling a cube, from which multiple elongated arms protrude symmetrically.

While these new morphologies represent an exciting frontier in perovskite NC research, there is no consensus yet concerning the factors that influence the shape evolution during growth. Moreover, the impact of these complex morphologies on charge carrier dynamics is yet to be explored. The specific morphology and flexibility of armed structures render them very attractive to act as building blocks for highly packed perovskite superstructures. The latter [13] have recently been shown to exhibit unique collective behaviors, for example, superfluorescence [14], amplified spontaneous emission [15, 16], and enhanced excitonic interactions [17]. However, most of the reported assemblies have been constructed from perovskite nanocubes [18, 19, 20, 21, 22, 23, 24, 25, 26, 27, 28, 29], which limit the possibility to tune optical responses and energy transport phenomena since interactions between cubic NCs are spatially uniform. In addition, nanocubes tend to form a simple cubic (SC) packing when self‐assembled. Armed structures, in contrast, present a flexible route to vary the 2D or 3D packing by controlling the size of the arms of the building blocks. Given the increasing interest in perovskite assemblies due to collective optical behavior and possible advanced photonic applications, a deeper understanding of such novel morphologies is timely and necessary, and it is important to systematically investigate the structure‐property connections of these newly emerging shapes.

In this study, we establish that arm length dictates both the optical properties of individual CsPbBr_3_ NCs and their self‐assembly behavior. We investigate the growth and 3D morphology of armed structures, identify the key factors controlling arm formation, and introduce synthetic routes that enable precise tuning of the arm length. Through ensemble and single‐particle optical measurements, we show that adjusting the arm length directly modulates charge‐carrier dynamics and emission characteristics. Finally, we show that arm length also influences the assembly formation, which, in turn, broadens the potential application space of these materials toward low‐threshold nanolasers, high‐brightness light‐emitting diodes (LEDs), high‐efficiency solar cells, and broadband photodetectors. We illustrate the importance of the armed structures as flexible building blocks for highly controlled and closely packed perovskite assemblies by investigating their configurations through 2D and 3D electron microscopy. In this manner, our findings offer new insights into the morphological diversity of CsPbBr_3_ and possible pathways for engineering next‐generation perovskite‐based materials and devices.

## 2 Results and Discussion

CsPbBr_3_ armed nanostructures were synthesized using a modified protocol based on the method reported by Pradhan et al. [12]. Unlike the conventional hot‐injection approach, where Cs‐oleate is typically introduced at elevated temperatures (140°C–200°C) [6], the method involves the injection of Cs‐oleate at a significantly lower temperature (18°C). This first synthesis step yields CsPbBr_3_ nanoseeds, which are subsequently purified by centrifugation to remove excess ligands, unreacted precursors, and byproducts. The purified seeds are redispersed in 1‐octadecene (ODE) and then injected into a hot, ligand‐free ODE solution at 245°C (synthesis step two).

As shown by the high‐angle annular dark‐field scanning transmission electron microscopy (HAADF‐STEM) overview image in Figure S1a, the first synthesis step results in the formation of nanoclusters with an average diameter of 2.5 ± 0.3 nm (Figure S1a,b), exhibiting a deep‐blue photoluminescence (PL) peak centered at 439 nm (Figure S1c). Selected‐area electron diffraction (SAED) pattern, such as in Figure S2a, indicates discernible diffraction rings, albeit broadened, which we attribute to the ultrasmall size of the clusters. The observed ring positions are consistent with the orthorhombic CsPbBr_3_ structure (*Pbnm*),  indicating that the seeds possess at least partial crystallinity rather than being fully amorphous. In addition, the non‐uniform intensity distribution along the rings suggests the possible presence of preferred orientations, allowing us to hypothesize the oriented attachment of the seeds during subsequent growth. These clusters show pronounced instability, especially in such common solvents as hexane and toluene, where they quickly agglomerate and exhibit a fast emission shift to green. Therefore, immediate progression to the next synthetic step is necessary, and for short‐term storage (<10 min), the nanoclusters are kept in ODE at low temperature (4°C). In contrast to previous observations by Pradhan et al. [12], where armed morphologies required a third post‐injection etching step with oleylammonium bromide on 26‐faceted rhombicuboctahedra, formed in the second stage, our synthesis yields CsPbBr_3_ armed nanostructures directly upon seed injection into hot ODE (245°C).

To elucidate the morphological evolution pathway from seeds to armed structures, we investigated intermediate species collected immediately after the injection of seeds (0 min, second stage of synthesis). HAADF‐STEM revealed the presence of the nanocubes in the orthorhombic phase (*Pbnm*, Figure S2b) with an average size of 10.1 ± 1.4 nm (Figure S1d,e) as reaction intermediates, exhibiting green emission at 518 nm with a photoluminescence quantum yield (PLQY) of 78 % (Figure S1f). Notably, no rhombicuboctahedral intermediates were observed at this step, suggesting an alternative growth pathway in comparison to [12], leading after 3 min of the second reaction stage to the formation of armed nanostructures of 31.2 ± 2.2 nm (Figure S1g–i), which includes the size of the central cube and the length of two arms. Since the central cube size for the final product was found to be 19.8 ± 2.6 nm (Figure S1g and Table S1), we conclude that first the cubic core was formed and then both arms and the cubic cores grew within 0–3 min of the reaction.

**FIGURE 1:**
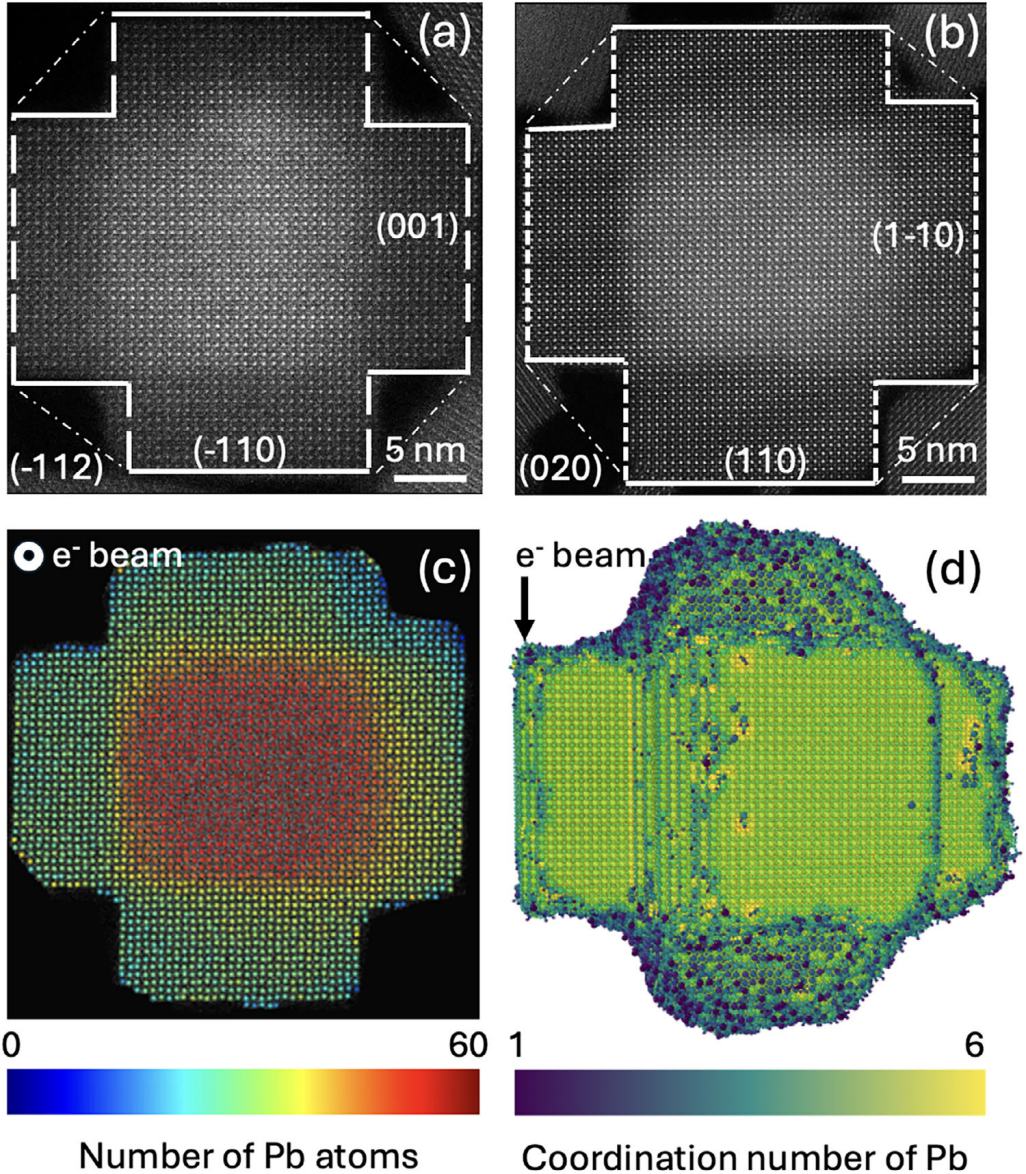
High‐resolution HAADF‐STEM images (a,b) of single CsPbBr_3_ armed structures imaged along the [110] and [001] zone axes (orthorhombic crystal system, *Pbnm*). Color‐coded map (с) depicting the distribution of the number of Pb atoms in each atomic column for the particle shown in (b). 3D atomic structure of the armed NC after structural relaxation (d).

Typically adopted under ambient conditions CsPbBr_3_ orthorhombic crystal structure (*Pbnm*) was confirmed in the synthesized armed nanostructures via HAADF‐STEM and SAED in Figure 1a,b, and Figures S2c and S3. A detailed crystallographic analysis revealed that the arms grow preferentially along four <110> and two <001> directions of the orthorhombic lattice (Figure 1a,b; Figure S4), whereas the {020} and {112} facets were notably absent as seen from [001] and [110] zone axes, respectively. When referenced to the idealized cubic perovskite crystal structure (*Pm‐3m*), the missing facets (Figure 1a,b, dash‐dotted lines; Figure S4 orange and green planes) correspond to a single {110}_c_ family (Supporting Information, calculation of the facets): specifically, the (020)_o_ and (−112)_o_ facets of the orthorhombic phase align with the (−110)_c_ and (−101)_c_ facets respectively. This suggests that arm growth occurs along directions where the {110}_c_ family of facets is not preserved (Supporting Information, calculation of the facets).

To further elucidate the 3D structure of the armed NCs, we reconstructed a 3D model from a single HAADF‐STEM projection. The intensity variations in the HAADF‐STEM images (Figure 1a,b) arise mainly from the thickness variations across the nanostructure. Hence, atom‐counting, based on statistical parameter estimation [30], was performed for one of the particles (Figure 1b). Next, the atom counts together with the positions of the atomic columns (Figure 1c) were used as input for a molecular dynamics (MD) simulation to obtain a relaxed 3D structure. As illustrated in the atomic 3D model of the obtained structures (Figure 1d; Movie S1), the arms indeed exhibit uniform growth along all six directions, consistent with the observed HAADF‐STEM projections (Figure S1g).

In conditions of ligand deficiency and elevated temperature (245°C), NC growth is more likely to be kinetically controlled instead of thermodynamically driven. Thermodynamic growth typically favors compact and highly symmetric morphologies [31], such as cubes or rhombicuboctahedra, minimizing the total surface energy. For instance, when seeds were injected into ODE in the presence of ligands (0.5 mL oleic acid and 0.5 mL oleylamine), predominantly polydisperse cubes/rectangular shapes were obtained (Figure S5). In contrast, under ligand‐free conditions, kinetic control can lead to more anisotropic shapes [31], as certain crystallographic facets advance more rapidly than others. We here observe such growth for the elongated arms that branch out from a core in four <110>_o_ and two <001>_o_ directions. To understand which synthetic parameters are crucial for the formation of this thermodynamically unstable morphology, the influence of key experimental variables, that is, seed concentration, seed size characteristics, and seed injection temperature, on the morphology of CsPbBr_3_ NCs, was investigated.

To elucidate the influence of seed concentration with respect to the final NC morphology, two batches of CsPbBr_3_ seeds were prepared at concentrations of 0.03 M (using the entire yield from the first synthesis step) and 0.003 M (10 times dilution). At the higher seed concentration, the resulting NCs predominantly exhibited a cubic morphology with an average size of 19.9 ± 2.1 nm (Figure S6a,b). In contrast, injection of the diluted seed solution led to the formation of the armed structures (Figure S6c,d). This observation suggests that at elevated seed concentrations, a dense distribution of nearby seeds supports uniform, more isotropic growth by quickly filling in reactive sites, including corners, and maintaining symmetric expansion. At lower seed concentrations, however, the reduced proximity between seeds allows unbalanced or facet‐selective extension, which promotes more anisotropic growth through directional agglomeration and oriented attachment, resulting in the formation of armed structures. As can be concluded from the sizes (19.9 ± 2.1 nm for the nanocubes (Figure S6b) vs. 19.8 ± 2.6 nm for the central cube dimensions in the armed structures (Table S1)), only the core is growing in case of concentrated seed solution injection, while with the seeds dilution, the arms formation from these cores is observed.

In parallel, we investigated the impact of seed size characteristics by synthesizing CsPbBr_3_ seeds at 40°C instead of 18°C (Figure S7a). The size analysis revealed a contribution of larger, agglomerated seeds (Figure S7c,d, dashed arrows), which are challenging to separate completely, thereby broadening the distribution from 1.5–3 nm (Figure S7b) to 1.5–6.5 nm (Figure S7d). Upon injection into hot ODE (245°C), these polydisperse seeds gave rise to a heterogeneous mixture of morphologies, including random irregularly shaped cubes and rectangles (Figure S7e,f, white dashed rectangles). These results suggest that altered seed characteristics, namely increased seed size together with the broader size distribution, compromise NCs growth uniformity.

We finally explored the role of injection temperature on final particle shape. At 245°C, the synthesis consistently produced well‐defined armed nanostructures (Figure S8a), indicative of kinetically dominated growth. When the injection temperature was reduced to 225°C, a mixture of cubic particles (Figure S8b, white dashed circles) and armed structures was formed. These findings suggest that lower temperatures slow down the reaction kinetics, enabling more controlled, more isotropic crystal growth and favoring equilibrium shapes. However, even under these conditions, there is some degree of facet‐selective growth, allowing the partial formation of non‐equilibrium (armed) morphology.

**FIGURE 2:**
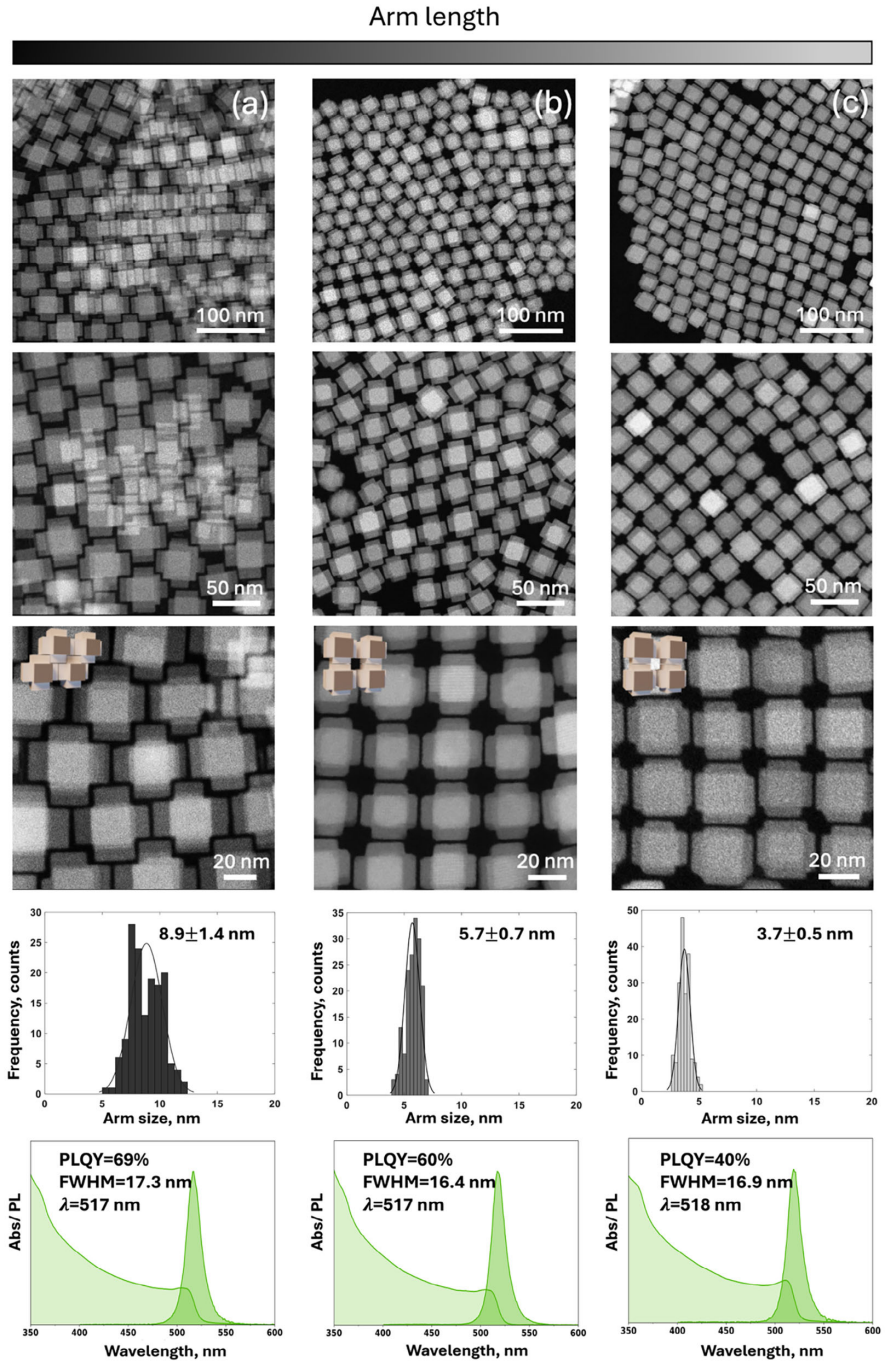
HAADF‐STEM overview images of (a) long‐armed, (b) middle‐armed, and (c) short‐armed structures with the corresponding arm length distributions and absorption/PL measurements. The arm lengths were achieved through the preparation with 0.3 mL Cs‐oleate (a), 0.2 mL Cs‐oleate (b), and as a result of storage of (b) in toluene in air for 24 h (c).

Based on these experiments, we conclude that the morphology of CsPbBr_3_ NCs is highly sensitive to seed size characteristics, seed concentration, and seed injection temperature (Figure S9). Next, we investigated how the arm length of the structures could be tuned without compromising the overall morphology and phase purity. A previous report [12] suggested that this goal could be achieved by adjusting the seed injection temperature. However, as discussed above, lowering the injection temperature to 225°C led to the coexistence of armed and cuboidal morphologies (Figure S8b). Instead, we focused on modifying the concentration of Cs‐oleate. Interestingly, a similar role of Cs^+^ concentration in modulating NC morphology was observed by Bera et al. [11] where annealing‐induced Cs^+^ and Br^−^ loss led to the transformation of 12‐faceted dodecahedral CsPbBr_3_ into 26‐faceted polyhedra due to changes in facet growth. Although the morphological outcome differs, our observations also suggest that Cs^+^ concentration critically influences facet‐selective growth, likely by altering surface passivation. Since the CsPbBr_3_ formation reaction is limited by the concentration of Cs^+^, increasing the volume of Cs‐oleate to 0.3 mL might change the surface coverage and passivation dynamics of the seeds, potentially causing certain facets to grow faster at the second step, hence, promoting the arm elongation. This results in a longer (8.9 ± 1.4 nm) arm length (Figure 2a), further called the long arm case, in comparison to the length achieved with 0.2 mL of Cs‐oleate (5.7 ± 0.7 nm—Figure 2b), further called the middle arm case. To quantify the morphological changes that occur to the NCs with Cs‐oleate volume change, we measured the ratio of the arm lengths vs. the sizes of the central cube (core) for long and middle arm cases (Figure 2a,b; Figures S1g and S10a and Table S1). Such calculations indicate that the arm‐to‐core size ratio decreased from 0.39 ± 0.07 to 0.29 ± 0.04 (Figure 2a,b). Further reduction of the Cs‐oleate volume to 0.1 mL yielded large (100–150 nm) particles with multiple short irregular arms (Figure S11). Interestingly, we discovered an alternative route to reduce the arm length of CsPbBr_3_ armed structures by simply storing NCs with middle arm lengths (Figure 2b) in toluene under ambient conditions (RH ≈ 40 %) at 20°C. A pronounced morphological instability was observed for these particles in toluene, in contrast to their behavior in hexane. Within 24 h, the arms began to exhibit smoother (Figure S12b), more rounded edges, resulting in a reduced arm‐to‐core size ratio of 0.18 ± 0.03 (Figure 2c; Figure S10b and Table S1), further called the short arm case. After three days of aging in toluene under ambient air, previously absent {020}_o_ and {112}_o_ facets began to emerge (Figure S12b,c), indicative of morphological evolution. Quantitative shape analysis, similar to the approach used to obtain the 3D structure in Figure 1d, confirmed the formation of 26‐faceted rhombicuboctahedra as a result of 3 days of storage of armed structures with the middle length in toluene exposed to the air (Figure S13 and Movie S2). Two possible contributors to this transformation are discussed in the caption of Figure S12: different polarity indices of toluene (p = 2.4) and hexane (p = 0.1) [32], and relatively high hygroscopicity of toluene (500 mg/L of water) compared to hexane (9.5 mg/L of water) [32]. In order to investigate the short‐armed structures before their degradation into 26‐faceted rhombicuboctahedra, the centrifugation until complete precipitation and subsequent redispersion of NCs in hexane, where they remain stable for at least one month, was performed.

The ability to differentiate the arm length offers an opportunity to systematically tune the optoelectronic properties of CsPbBr_3_ NCs, which define potential applications of these materials. We therefore first assessed arm‐length‐dependent optical behavior using steady‐state measurements. Remarkably, almost no shift in the excitonic peak position was observed in the steady‐state absorption spectra for the NCs with varying arm lengths, indicating no change in bandgap energy (Table S2). Similarly, the PL emission maxima remain unchanged at 517–518 nm across all samples (Figure 2). However, some variation in PLQYs was detected depending on the arm length (Table S2 and Figure 2). The NCs exhibited PLQYs of 69 %, 60 %, and 40 % for the long‐, middle‐, and short‐arm cases, respectively. Nevertheless, these measurements are done in the colloidal state and provide ensemble‐averaged information. Intricate details of the optoelectronic response of individual particles are lost due to signal averaging or dynamic interparticle interactions, providing a convoluted picture in these steady‐state measurements. In contrast, investigating time‐resolved PL response at the single particle level, such as PL blinking and PL decay, can reveal the prospect of the tunable optoelectronic response of these armed CsPbBr_3_ NCs.

**FIGURE 3:**
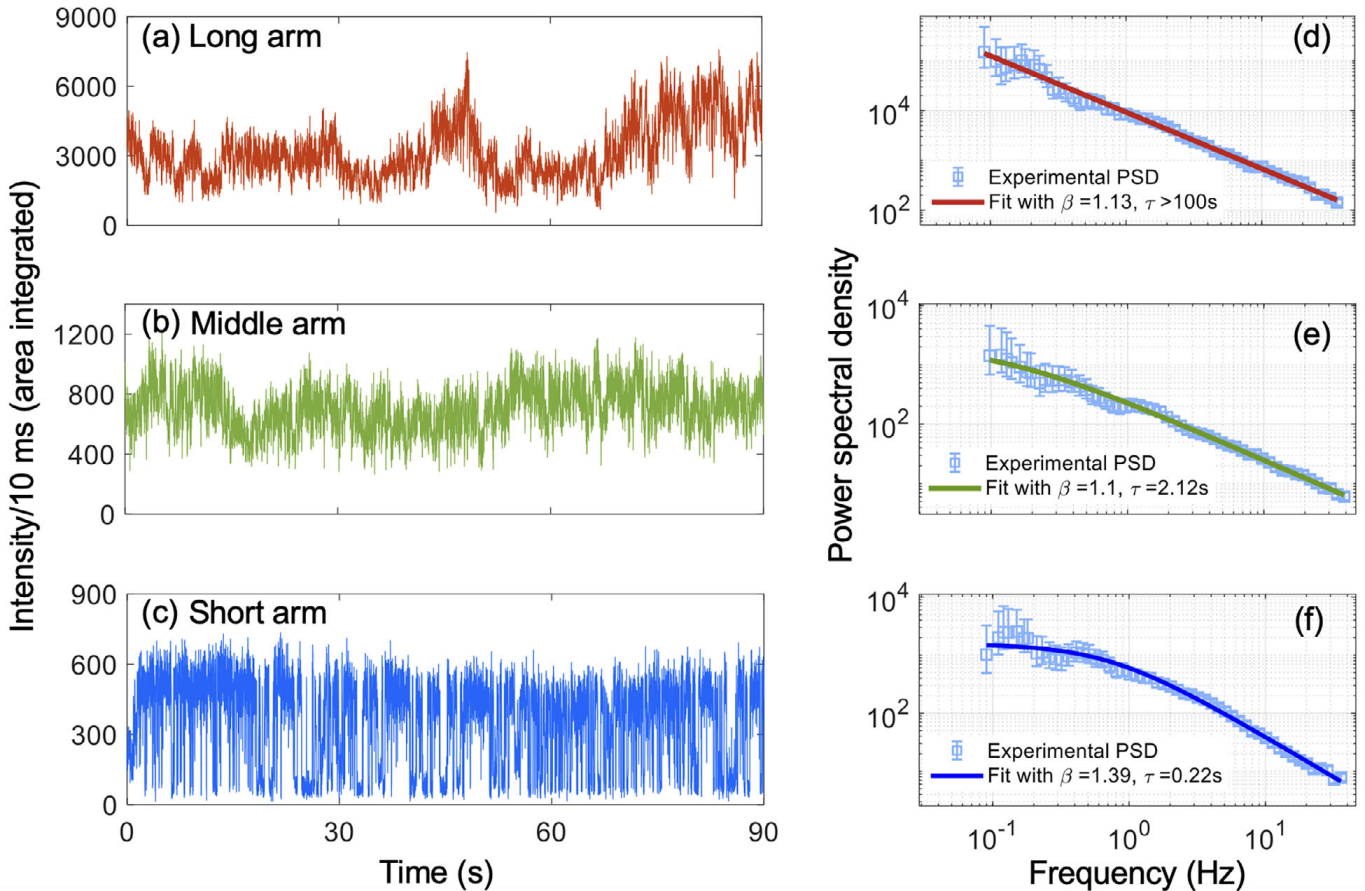
Single‐particle PL fluctuation results for long (a), middle (b), and short (c) armed structures with the corresponding power spectral density plots (d,e,f). PSDs follow a power law (*A*/*f* ^β^, long arm) or stretched Lorentzian behavior ($A^{\#}/\left(1+{\left(f/f_{0}\right)}^{\beta }\right)),$where τ  =  1/2π*f*_0_, middle and short arm).

PL blinking, a universal phenomenon of single emitters [33], including quantum dots and NCs, reflects underlying charge carrier and defect dynamics, which are critical to understand their optoelectronic performance [34]. It originates from the occasional charge trapping and subsequent nonradiative recombination through metastable surface trap states. Despite consistent steady‐state optical behavior, single‐particle PL dynamics differ markedly for three different arm lengths (Figure 3a–c). About 60 individual particles of each type of NCs were studied, and representative results are discussed below. As the arm length decreases, the PL behavior evolves from intensity flickering to pronounced blinking characterized by abrupt on–off transitions [35]. Notably, long‐ and middle‐armed NCs maintain a finite emissive state and never fully transition to the dark state (off), in contrast to short‐armed NCs, which exhibit complete PL quenching to a dark state (Figure 3a–c; Figure S14). We will use the term PL fluctuation to describe the flickering and blinking behavior of these NCs. Furthermore, under the same experimental conditions, the highest intensity in the respective fluctuation time traces decreases with a decrease in arm length, having an order of magnitude difference between the long arm (6000 counts/10 ms) and the short arm (600 counts/10 ms). This suggests that trap‐mediated PL quenching is more prominent for the short arm case compared to longer armed NCs (long and middle arm cases).

To obtain further insights into the transient charge trapping processes, we performed statistical analysis of intensity fluctuation events in time traces (Figure 3d–f). Since the traditional bin & threshold method for obtaining probability distribution of on and off events is inapplicable for such flickering systems, we implemented the power spectral density (PSD) estimation method [36, 37]. Depending on the fluctuation type, the shape of PSD can range from a Lorentzian profile, signifying a specific fluctuation center with a defined switching rate, to a power‐law having dispersive switching rates. Such behavior is consistent with the multiple recombination center (MRC) model of PL fluctuation [38, 39, 40]. As shown in Figure 3d–f, PSDs revealed a systematic transition from power law behavior (*A*/*f* ^β^) observed for long arms to stretched Lorentzian ($A^{\#}/(1+{\left(f/f_{0}\right)}^{\beta }$)) kinetics observed for the short arm case, indicating a shift from dispersive fluctuation kinetics to distinct two‐level switching with a characteristic timescale (τ = 1/2π*f*_0_, in Figure 3d–f). Here *A*, $A^{\#}$ are the amplitude of PSD and β is the exponent. PSD plots for long‐armed NCs demonstrate the distribution of transient PL quenching rates over at least four orders of magnitude in time. For the middle‐armed NCs, PSDs have a tendency to saturate at low frequencies, while there is a clear Lorentzian saturation in the case of short‐armed NCs, meaning that one specific type of trap (with a characteristic timescale, τ) dominates the transient PL quenching process. However, the PSD slope for the short arm case (β ≈ 1.42 ± 0.08) at high frequencies deviates from the ideal Lorentzian (β = 2), indicating contributions from additional fast fluctuation processes. A change of the PSD slope from 1.42 to 1.12 ± 0.04 and 1.16 ± 0.01 as observed for middle and long arms, respectively, indicates an increasing distribution of PL fluctuation rates.

**FIGURE 4:**
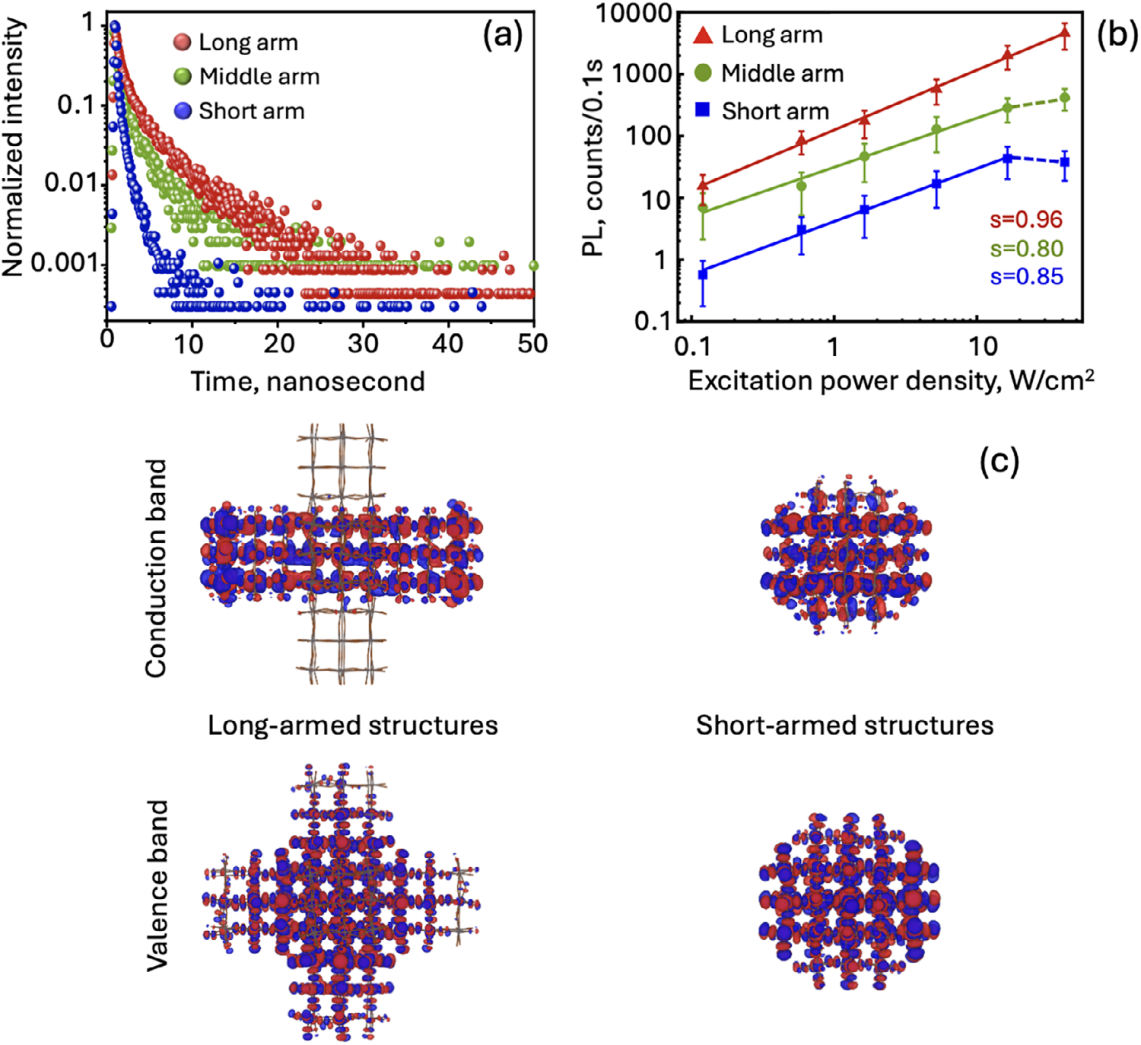
Time‐resolved PL decays for all three arm lengths (a). Excitation power density‐dependent PL of different armed structures (b). S in (b) corresponds to the slope of the linear part of the plot for long, middle, and short arm cases. Isosurfaces of the molecular orbitals (MO) at the CB and VB edges (c) computed at the DFT level with a counter value of 0.02 e Bohr^−3^, highlighting positive and negative parts in red and blue, respectively. MO plots in (c) indicate an increased delocalization of both electron and hole wavefunction for long‐armed CsPbBr_3_ NCs (left) compared to short‐armed CsPbBr_3_ NCs (right).

To extract further insight into the charge carrier and defect dynamics, we have performed time‐resolved PL measurements on several single particles (∼20 for each sample). PL decays also show a systematic change with the arm length. As the arm length decreases, the PL decay becomes faster (Figure 4a). All decay curves fit well to a biexponential function, with both lifetime components increasing with arm length (Table S3 and Figure S15). Based on previous literature reports on single CsPbBr_3_ NCs, the subnanosecond component can be assigned to the trion recombination and the longer component to the excitonic recombination [35]. Trions are systems of one charge plus an exciton, which originate from trapping of the counter charge in a long‐lived surface state [41], and are susceptible to the Meitner–Auger process in which exciton recombination energy is non‐radiatively transferred to the third charge or recombines radiatively with a faster rate than a single exciton. Assuming that armed nanostructures act as single‐photon emitters, where only one exciton is present per particle under low excitation conditions, we can consider the role of trions in the carrier recombination dynamics. This assumption is supported by previous reports, which show that CsPbBr_3_ NCs of similar dimensions to the current work (core size of ∼20 nm for all three arm lengths, Table S1) exhibit single‐photon emission behavior [42, 43]. For the short arm case, faster subnanosecond decay with larger amplitude (Table S3) likely implies more efficient trion recombination, which is Meitner–Auger‐dominated. This is consistent with the hypothesized larger number of surface traps (arising from the short‐armed NCs preparation route (Figure S12)), which promote the formation of these trions. In addition, faster excitonic decay (the longer component, Table S3) observed for short‐armed NCs as well indicates increased nonradiative pathways even for neutral excitons, presumably due to more surface defects.

As the next step in the investigation of carrier recombination mechanisms, we have performed excitation power‐dependent PL measurements for the three types of samples. As shown in Figure 4b and Figure S16, the PL intensity increases linearly as the excitation power density of a continuous wave laser increases from 0.1 to 40 W/cm^2^. Whereas for the long‐armed structures a slope close to unity (s ≈ 1) is maintained across the entire power range, the response for the medium‐ (s ≈ 0.8) and short‐armed (s ≈ 0.85) structures begins to sub‐linearize above ∼10 W/cm^2^. A linear increase at low excitation densities indicates that single‐exciton recombination dominates below this threshold value (i.e., below 10 W/cm^2^). Deviation from linearity at higher power densities (i.e., above 10 W/cm^2^) for middle‐ and short‐armed structures is attributed to the nonradiative processes, primarily Meitner‐Auger recombination, which suppresses radiation recombination of multiple excitons [44, 45]. Although multiple excitons may be generated at high power densities in all structures, the preservation of linearity in the long‐armed structures suggests a reduced Auger recombination rate due to weaker carrier confinement. Furthermore, we measured PL blinking traces and constructed corresponding PSDs at 5 and 41 W/cm^2^ for long‐ and short‐armed structures (examples are given in Figures S17 and S18). The increase of heterogeneity in blinking events and the transformation of stretched Lorentzian PSD to near power law in short‐armed NCs at high excitation power suggest participation of additional intensity fluctuation events, likely, as already mentioned, due to enhanced Meitner‐Auger process. In contrast, no significant change in PL blinking behavior or corresponding PSD shapes has been observed in long‐armed structures upon excitation power density increase. We note, however, that the PSD of the long‐armed structures already exhibits a power‐law character at low excitation power; therefore, a moderate increase in Meitner–Auger processes is not expected to alter this signature noticeably.

Summarizing all experimental observations related to optical properties, we propose that the electron and hole wavefunction delocalization increases as the arm length increases, given that the arms are epitaxially grown over the core cube. Consequently, oscillator strength falls with an increase in the arm length, resulting in reduced nonradiative Meitner–Auger recombination through minimized carrier‐carrier and carrier‐exciton interaction. These facts are supported by the increase in PL intensity in the time traces, absence of complete dark states, and increase in PL lifetime components as the arm length increases (Figures 3a–c and 4a). To reveal how the arm length influences the electronic structure of CsPbBr_3_ NCs and further confirm the hypothesis about electron and hole wavefunction delocalization, we performed Density Functional theory (DFT) calculations. We built the armed nanostructure models from a cubic CsPbBr_3_ NC of about 1.8 nm and added rectangular CsPbBr_3_ arms with dimensions of 1.8 nm × 1.8 nm × H, where H corresponds to the lengths of 1 (0.6 nm), 2 (1.2 nm), or 3 (1.8 nm) unit cells. Based on these models, we computed the electronic structure and evaluated the participation ratio (PR) and inverse participation ratio (IPR) of the frontier orbitals, as reported in Figure 4c and Figure S19. In all three cases, both the valence band (VB) and conduction band (CB) edge states correspond to mixed arm–core configurations, with a progressively larger contribution from the arms as the arm length increases, as highlighted by the molecular orbital plots in Figure 4c. This leads to an increased delocalization of both electron and hole wavefunctions with arm length rise, further evidenced by the increase in PR values from 113 to 194 to 208 for the VB edge, and from 35 to 51 to 69 for the CB edge in the short‐, medium‐, and long‐armed CsPbBr_3_ NCs respectively.

The increase in the characteristic timescale of PL fluctuation (from 0.2 s to >100 s) and the decreasing power law exponent (Figure 3d–f) suggest increasing variation of interaction between charges and metastable surface traps due to spatial separation between the cubic core and the arm surface. Therefore, arms likely introduce a variable energetic barrier between charges/excitons and surface traps, depending on the arm length. A longer arm length results in reduced nonradiative recombination by diminishing Meitner‐Auger and MRC blinking/fluctuation. Given their superior optical properties, including high PLQY and suppressed blinking, long‐armed CsPbBr_3_ NCs are a promising material for applications in optoelectronics and quantum photonics, that is, for LEDs, lasers, and photodetectors. In contrast, short‐armed CsPbBr_3_ NCs, despite exhibiting lower radiative efficiency, demonstrate distinct advantages arising from their unique blinking characteristics, faster recombination kinetics, and enhanced surface accessibility. These properties make them suitable candidates for application in chemical sensing or for gated single photon output in high‐speed optoelectronic technologies such as quantum dot‐based qubits.

**FIGURE 5:**
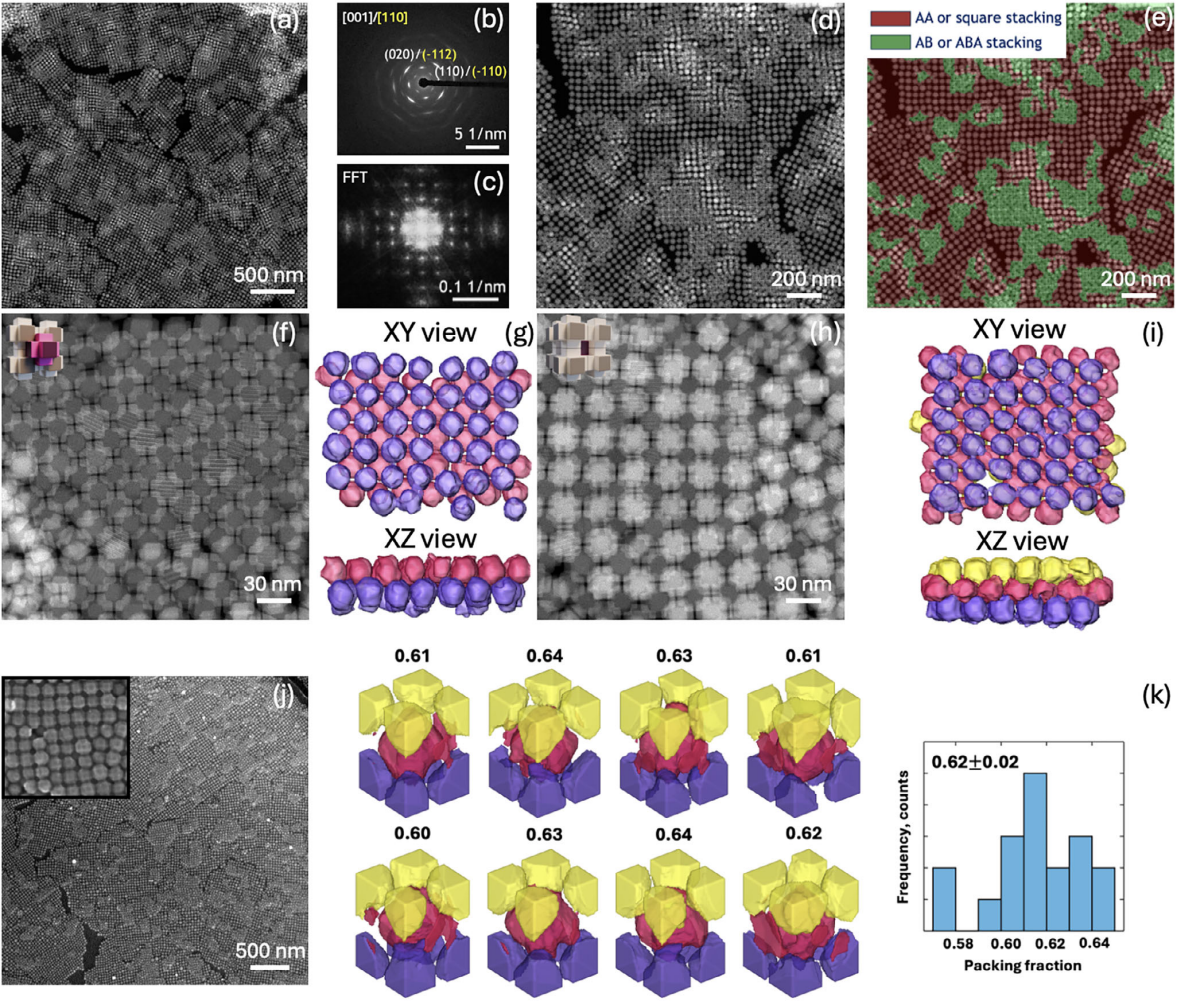
HAADF‐STEM overview image of the middle‐length armed structures assembled into 2D/3D superstructures (a). SAED and Fourier Transform (FT) patterns for the middle‐armed NCs assemblies (b,c). Color‐coded map (e) for HAADF‐STEM image (d) depicting the regions with either AA/square stacking (red) or AB/ABA stacking (green). HAADF‐STEM zoomed images for bilayer and triple layer regions (AB and ABA stacking) observed after assembling middle‐length armed structures (f,h). ET reconstructions of AB and ABA assembled regions shown in XY and XZ directions (g,i). Different colors in the reconstruction correspond to different NC layers. SEEBIC overview image (j) of the assemblies composed of the middle‐armed structures with an inset at higher magnification. Experimental packing fractions of medium‐armed structures (k). Representative subvolumes corresponding to individual BCC unit cells (ABA stacking) extracted from the ET reconstruction of the 3D superstructure are shown together with a histogram depicting all packing fractions measured for 18 BCC unit cells.

Morphology, and in particular arm length, is critical not only for defining the optical properties but also for governing the assembly behavior. The possibility of assembling the NCs expands the potential applicability of these materials toward collective‐emission devices, high‐efficiency solar cells, and broadband photodetectors. From TEM investigations performed during the synthesis optimization, it became evident that CsPbBr_3_‐armed nanostructures possess a strong tendency to self‐assemble upon solvent drying, with the arm length playing a decisive role in determining the resulting packing geometry. For instance, we noticed a tendency for NCs with long arm lengths to form ordered monolayer regions with centered rectangular packing (*cr*), the plane group *cmm* (Figure 2a). In case of reduced arm length to the middle size, there was a distinct change in the packing of the NCs in a monolayer from centered rectangular to a square arrangement, that is, a primitive (*sp*) lattice with the plane group *p4mm* (Figure 2b). For the short‐armed NCs, we have also observed a square (*sp*) lattice arrangement in a monolayer (Figure 2c). Based on this pronounced and reproducible behavior, the solvent drying technique was chosen as the primary method to prepare and study NCs assemblies throughout this work [13, 46, 47, 48, 49]. Surprisingly, while such assemblies could be obtained for building blocks with the middle and short arm length, this was not feasible for the long‐armed structures. We attribute this observation to the inherent layer‐by‐layer growth mechanism of assembling during solvent drying, wherein an initial close‐packed long‐armed monolayer forms with a centered‐rectangular symmetry (Figure 2a), but subsequent layers exhibit a random disordered arrangement because of the lack of possibility for the NCs with such long arms to move and stack in 3D. For the middle‐armed NCs, we achieved large‐area multilayer superlattices with lateral domain sizes exceeding 500 nm, as shown by HAADF‐STEM (Figure 5a). In these assemblies, the NCs are atomically aligned along [001] and [110] directions of the orthorhombic lattice (Figure 5b). A detailed analysis by HAADF‐STEM (Figure 5d,e) identified two distinct stacking configurations in 3D, both exhibiting a superlattice periodicity of approximately 33 nm (Figure 5c): an AA stacking resembling a simple cubic (SC) superlattice (Figure 5e, red) and an AB/ABA stacking resembling body‐centered cubic (BCC) superlattice (Figure 5e, green). To enable direct visualization of the number of stacked layers and reveal the overall spatial arrangement and connectivity of the arms within the assembled superstructures, we performed electron tomography (ET), which confirmed that these assemblies typically consist of two to three layers with the proposed stacking motifs (Figure 5f–i; Movies S3 and S4). Topographical information in TEM for these assemblies was additionally accessed through secondary electron beam‐induced current (SEEBIC) images (Figure 5j; Figure S20a,b). Based on ET reconstruction results, we were able to isolate individual supercells within the assemblies and determine their experimental packing fractions. By extracting the 3D contour of each supercell and calculating the volume occupied by the constituent NCs, we obtained packing fractions of 62 ± 2 % (Figure 5k).

**FIGURE 6:**
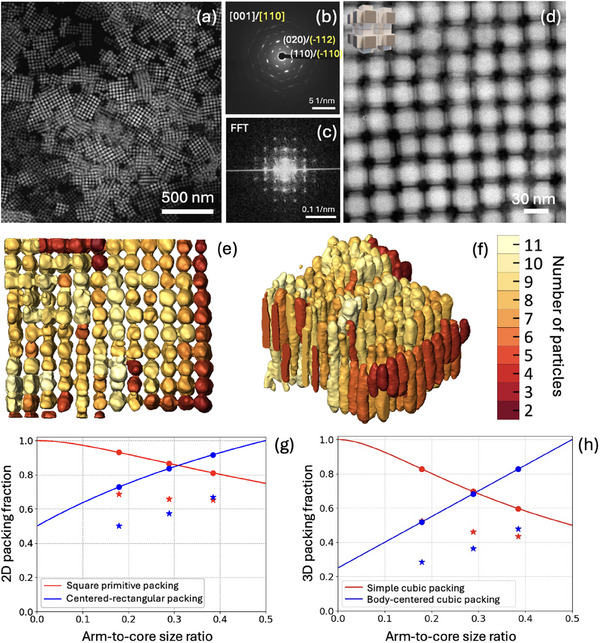
HAADF‐STEM overview image of the short‐length armed structures assembled into 2D/3D superstructures (a). SAED and FT patterns for the short‐armed NCs assemblies (b,c). HAADF‐STEM zoomed image for multilayer regions (AA stacking) found after assembling short‐length armed structures (d). ET reconstruction of AA stacked region with a color‐coded map showing the number of armed particles in a z‐direction (e,f). The size of each particle is assumed to be ∼30 nm. Theoretical 2D (g) and 3D (h) packing fractions corresponding to the square arrangement of the NCs in a monolayer and SC superlattices (red line), respectively, as well as centered rectangular arrangement in a monolayer and BCC superlattices (blue line) ccordingly for different arm‐to‐core size ratios. Stars/circles highlight the packing densities for experimentally observed ratios with/without ligand consideration, respectively.

In contrast, short‐armed NCs formed morphologically distinct assemblies (Figure 6a). NCs are also atomically aligned along [001] and [110] directions of the orthorhombic lattice (Figure 6b); however, only an AA‐type of 3D stacking was observed (Figure 6d) for these NCs with SC superlattice dimensions of approximately 30 nm (Figure 6c). Another difference in comparison to middle‐armed NCs is that the resulting films exhibited lower thickness uniformity with localized regions containing multiple NC layers along the z‐axis and with an average domain size of 200 nm. Despite the limited resolution along the electron beam direction inherent to ET reconstruction, knowledge of the average size of individual short‐armed NCs enabled us to estimate that the maximum number of vertically stacked layers can reach up to 11 (Figure 6e,f; Movie S5).

The observed difference in the dimensions of resulting assemblies for short‐ and middle‐armed NCs is likely driven by variations in solvent evaporation dynamics–toluene vs. hexane–reflecting the influence of solvent boiling point on assembly kinetics. Collectively, these results highlight the tuneability of armed NC assemblies via control over particle geometry and solvent environment, offering new pathways for engineering 3D perovskite superstructures with tailored optoelectronic properties.

Control over NC packing in perovskite assemblies is essential because it directly governs interparticle electronic and optical coupling. Long‐range order plays a critical role, as periodic arrangements enable coherent excitonic coupling, reduce optical scattering, and allow the emergence of collective phenomena such as superradiance [14, 15, 16]. In addition to order, a high packing density further strengthens interparticle interactions by reducing the interparticle spacing, enhancing wavefunction overlap, and promoting efficient exciton and charge transport [50, 51]. To further assess the structural compactness of the armed CsPbBr_3_ NC superlattices, we calculated their packing fractions as a function of the arm‐to‐core size ratio (Figure 6g,h, Supporting Information Calculation of packing densities) for monolayers and 3D self‐assemblies. First, we considered a monolayer arrangement of armed structures either in a centered rectangular or square primitive geometry (Figure 6g). In the long arm case, the packing fraction is maximized by adopting a centered rectangular packing (92 % for *cr* vs. 81 % for *sp*, Table S4), whereas in the middle and short arm cases, the arrangement in a square monolayer is spatially more efficient (packing fractions of 87 % and 93 % for *sp* vs. 84 % and 73 % for *cr* respectively, Table S4), which is in line with the morphological selectivity we observe experimentally (Figure 2). We note that these trends are preserved upon inclusion of organic surface ligands at the surface of the NCs, which introduce interparticle steric hindrance, thus reducing the computed packing fraction in both centered‐rectangular and square 2D assemblies (Table S4; blue and red stars in Figure 6g). Next, we considered the 3D arrangement of armed CsPbBr_3_ NCs either in SC (*Pm‐3m*) or BCC (*Im‐3m*) 3D assemblies (Figure 6h). For the middle‐armed 3D assemblies, both SC and BCC superlattices exhibited comparable theoretical packing fractions of 70 % and 68 %, respectively (Table S5). These results align well with our experimental observations (Figure 5), where both stacking types were present, reflecting their similar spatial efficiencies. Importantly, shorter arms facilitated significantly higher packing densities. The values reached 83 % in SC superlattices, which is higher in comparison to the BCC configuration for the same arm length (52 %, Table S5). In this case, the SC configuration was the only one observed experimentally (Figure 6a,d–f). When the presence of organic surface ligands (blue and red stars in Figure 6h) was taken into account, the packing fraction of the short‐armed SC superlattice decreased to 53 %, whereas the values for the middle‐arm SC and BCC superlattices dropped to 46 % and 37 %, respectively. As noted earlier, ET reconstruction enabled quantitative determination of the packing fraction in the middle‐armed BCC superlattices. The measured value of 62 % lies between the packing fractions predicted with a maximal amount of ligands inducing the longest stretching as well as without any ligand contribution and is, therefore, fully consistent with the theoretical expectations. Overall, these results showcase how the symmetry of both 2D and 3D superlattices depends on the arm‐to‐core size ratio and how these symmetries are dictated by the criterion of maximizing packing efficiency. Both NC packing motifs and efficiency, tunable via arm length, offer a promising structural advantage for constructing dense, ordered perovskite films with potential optoelectronic benefits.

## 3 Conclusion

In this study, we explored the synthesis, structure, and optoelectronic properties of morphologically tunable CsPbBr_3_ NCs known as armed structures, offering a new dimension of morphological diversity in perovskite NC research. Through a detailed synthetic investigation, we identified three key parameters essential for obtaining morphologically pure armed NCs: seed size characteristics, seed concentration, and the temperature at which seeds are injected. We found that Cs‐oleate concentration serves as an effective lever for controlling the arm length of these structures. In addition, we discovered that prolonged storage of these NCs in toluene under ambient conditions induces a gradual transformation of armed structures into 26‐faceted rhombicuboctahedra, with a short‐armed intermediate morphology forming as a transitional state. This allowed us to isolate and systematically study three distinct samples with different arm lengths.

Comprehensive ensemble and single‐particle optical characterization, including steady‐state PL, PL blinking, excitation power‐dependent PL, and time‐resolved PL decay, revealed a clear dependence of optoelectronic properties on arm length. As the arm length increased, we observed enhanced radiative efficiency, longer PL lifetimes, elimination of complete dark states, and significant suppression of blinking. These properties make long‐armed NCs excellent candidates for optoelectronic and quantum photonic applications such as LEDs, lasers, and photodetectors. At the same time, the short‐armed NCs demonstrated distinct advantages such as faster recombination kinetics, pronounced PL blinking, and enhanced surface accessibility. These characteristics, in turn, make them promising for chemical sensing, as well as for gated single‐photon emission applications in high‐speed optoelectronics and quantum technologies.

Beyond individual NCs' optical behavior, we demonstrated that armed structures can be assembled into large‐scale superlattices with the arm length modulating the resulting geometry. Using 2D and 3D electron microscopy, we fully characterized the assembled architectures and quantified their packing fractions.

Overall, our findings show that arm length tuning provides a direct route to control both the optical response of individual CsPbBr_3_ NCs and their ordering tendencies. By demonstrating how a single geometric feature governs emission behavior and superlattice formation, we highlight the potential of armed CsPbBr_3_ NCs as versatile building blocks for next‐generation optoelectronic and photonic materials.

## 4 Experimental Section

### 4.1 Synthesis

#### 4.1.1 Chemicals

Cesium carbonate (Cs_2_CO_3_, 99.9 %, Aldrich), lead(II) bromide (PbBr_2_, 99.999 %, Aldrich), oleic acid (OA, 90 %, Aldrich, freeze‐dried), oleylamine (OLA, 80 %–90 %, Acros Organics, freeze‐dried), 1‐octadecene (ODE, 90 %, Aldrich), toluene (anhydrous, 99.8 %, Aldrich), hexane (anhydrous, 97 %, Acros Organics).

#### 4.1.2 Cs‐Oleate Preparation

Cs‐oleate precursor was synthesized following the established procedure reported by Protesescu et al. [12]. Specifically, 0.652 g of Cs_2_CO_3_ and 17.5 mL of 1‐octadecene (ODE) were loaded into a 100 mL three‐neck flask and degassed under vacuum. Subsequently, 2.5 mL of oleic acid (OA) was injected, and the mixture was heated to 120°C under vacuum for approximately 1 h to ensure complete dissolution and complete reaction. The resulting clear Cs‐oleate solution was collected while hot under a nitrogen atmosphere and stored in a nitrogen‐filled vial to prevent degradation. Prior to use, the precursor was preheated to 100°C to redissolve any Cs‐oleate precipitate formed at room temperature.

#### 4.1.3 Seeds Preparation

CsPbBr_3_ seed clusters were synthesized following a previously reported procedure with minor modifications [12]. In a typical synthesis, 0.0734 g of PbBr_2_ and 3 mL of 1‐octadecene (ODE) were loaded into a 50 mL three‐neck flask and subjected to vacuum degassing. Afterward, 0.5 mL of oleic acid (OA) and 0.5 mL of oleylamine (OLA) were injected under a nitrogen atmosphere. The mixture was maintained at 120°C until a clear solution was obtained, indicating complete dissolution of PbBr_2_. The solution was then cooled to 18°C. Subsequently, 0.2 mL of pre‐heated Cs‐oleate solution was swiftly injected. After 20 min of reaction at this temperature, seed NCs were isolated via centrifugation at 10 000 rcf for 10 min. The resulting supernatant was discarded, and the precipitate was dissolved in 1 mL of ODE. The seeds were stored at 4°C and used shortly thereafter to ensure colloidal stability.

#### 4.1.4 Armed Structures Synthesis

A total of 3 mL of ODE was introduced into a 50 mL three‐neck flask, degassed under vacuum, and heated to 240°C–245°C under nitrogen flow. At the target temperature, 1 mL of previously prepared CsPbBr_3_ seed solution was swiftly injected. The reaction was allowed to proceed for 3 min before being quenched by immersion of the flask in an ice bath. The crude product was distributed into four 1 mL vials and centrifuged at 500 rcf for 3 min. The resulting supernatant was discarded, and the precipitate was redispersed in 200 µL of hexane. This solution was centrifuged again at 500 rcf for 1.5 min. The upper fraction, containing the colloidally stable armed NCs, was collected and used for further characterization or assembly.

#### 4.1.5 Preparation of Armed Structures Self‐Assemblies

CsPbBr_3_‐armed superlattices were fabricated via a drying‐mediated self‐assembly approach on carbon‐coated TEM grids. A 30 µL of CsPbBr_3_ NCs solution in hexane/toluene was transferred into a tilted 2 mL glass vial containing the substrate at the bottom. The solvent was slowly evaporated at 20°C under reduced pressure (0.2 atm) for approximately 24 h, allowing the formation of ordered NC assemblies.

### 4.2 Transmission Electron Microscopy (TEM) Imaging and Quantification

As‐prepared solutions of СsPbBr_3_ NCs were drop‐casted onto a carbon‐coated copper grid. The solvent was allowed to evaporate prior to imaging.

#### 4.2.1 HAADF‐STEM Imaging, Electron Diffraction, and Electron Tomography

HAADF‐STEM images were acquired using a probe‐corrected Thermo Fisher Titan Themis Z microscope operated at 300 kV with a probe convergence angle of 18–21 mrad. Electron diffraction (ED) patterns were acquired using a 200 µm selected area aperture. HAADF‐STEM image analysis was performed using ImageJ with the Trainable Weka segmentation plug‐in [52]. Electron tomography was also carried out in HAADF‐STEM mode. For that, HAADF‐STEM images at different tilt angles were manually recorded using a dedicated tomography holder (Fischione 2020) over ±65°–70°, with a tilt increment of 3°–5°. The image acquisition, as well as position correction and focusing, was performed at the beam currents of <30 pA to avoid the formation of Pb/PbX_2_ clusters at the NCs surface [53, 54]. All images acquired for tomography were corrected for noise and scanning artefacts using a neural network tailored for STEM image restoration [55]. Tilt series were then aligned with a combination of cross‐correlation and manual correction. Reconstructions were computed with the weighted back projection (WBP) or the expectation maximization algorithm as implemented in the Astra Toolbox (v. 1.8 for MATLAB). Segmentation was performed through 3D pixel classification with ilastik (v. 1.4.0) [56]. Three classes were selected for the background, interparticle space, and the particles themselves. All training features were selected with sigma 0.7, 1, and 5. The segmented particle maps were cleaned with a 3D morphological opening, and individual particles were isolated with a 3D distance transform watershed as implemented in ImageJ's MorpholibJ plugin, with Borgefors distance, normalized weights, 1.0 dynamic, and 6 voxels connectivity. For middle arm samples, the small thickness of the layers as well as particles’ arrangement allowed complete separation along the height direction, and the particles were color‐coded based on the height of their center of mass for visualization. For the short arm sample, the columns of the AA stacking were separated, and their height was measured by the length of their principal axis, calculated with MATLAB's regionprops3. To estimate the number of particles per column, an individual diameter of 30 nm was assumed. Visualization was done in Amira (v. 5.4.0). The experimental packing fractions from ET reconstructions were estimated on the population of particles that belonged to complete BCC unit cells. These were extracted by locating the center of mass of each individual particle and subsequently identifying the nearest neighbors. Volumes corresponding to one unit cell were extracted by cropping the cube formed by the centroids of the top 4 and bottom 4 particles.

#### 4.2.2 Quantification of 3D Models

For the 3D shape reconstruction of the atomic structure from a single HAADF‐STEM image, the positions of Pb atomic columns were determined by fitting 2D Gaussian functions through a nonlinear least‐squares optimization using the StatSTEM software [30]. The total scattered intensities were then quantified within Voronoi cells constructed around each fitted Pb atomic column [57, 58]. These total integrated intensities for each unit cell of the CsPbBr_3_ scale with the sample thickness (i.e., the number of unit cells along the beam direction). The distribution of the integrated intensities from all the unit cells was further decomposed into multiple overlapping components. The number and locations of these components were determined using a so‐called Gaussian mixture model analysis in combination with an order selection criterion [59, 60]. This order selection criterion determines the number of statistically significant components, which often corresponds to a minimum in the evaluation of this criterion as a function of the number of components. To accurately determine the number of components (Figure S21a,b), which in turn corresponds to the number of unit cells along the z‐direction (parallel to the beam), HAADF‐STEM image simulations were performed using the MULTEM software package [61, 62]. The integrated unit cell intensities derived from the simulations were compared with experimental data (Figure S21c,d). An acceleration voltage of 300 kV, a semiconvergence angle of 18–21 mrad, and a pixel size of 0.3 Å were chosen, and averaging over 30 unique phonon configurations was performed. The full set of simulation settings is listed in Table 1.

**TABLE 1:** Details of the HAADF‐STEM images simulations with the MULTEM software package.

Parameter	Value
Acceleration voltage	300 kV
Defocus	14.0312 Å
Spherical aberration	0.001 mm
Convergence angle	18–21 mrad
Inner detector angle	36–46 mrad
Outer detector angle	215–222 mrad
Root mean squared displacement Cs	0.2804 Å
Root mean squared displacement Pb	0.1764 Å
Root mean squared displacement I	0.2500 Å
Number of phonon configurations	30

The atom‐counting results were used as input for an energy minimization scheme to obtain a 3D model of the investigated structure. The initial 3D configuration was created by positioning the number of unit cells symmetrically around a central plane along the beam direction. The initial model of the nanoparticle was geometrically relaxed by minimizing the potential energy using a conjugate gradient algorithm to reduce the internal stresses and bring the structure to a local energy minimum. Then, molecular dynamics (MD) simulations were performed at 500 K in the canonical ensemble (NVT) using a Lennard‐Jones‐Coulomb potential [63] to thermally relax the structure, allowing surface atoms to reorganize and equilibrate.

#### 4.2.3 Secondary Electron Electron Beam Induced Current (SEEBIC) Imaging

SEEBIC imaging [64] was performed at an acceleration voltage of 200 kV and a beam current of 250 pA. A custom‐made transimpedance amplifier with a first‐stage gain of 4 × 10^8^ V/A and a bandwidth of 4 kHz, electrically connected to the sample via a DENS Solutions Wildfire holder, was used to convert the SEEBIC signal into a voltage signal digitized by the Attolight OUDS II scan engine. SEEBIC images of 1024 × 1024 pixels were acquired with a dwell time of 250 µs. We used a TEM grid carrier based on a 0.6‐mm thick FR4 printed circuit board, designed to fit in a DENS Solutions Wildfire heating holder. To minimize the effect of beam damage and carbon contamination, sample navigation and image focusing were done at reduced electron doses (beam current < 30 pA).

### 4.3 Density Functional Theory (DFT) Calculation

Atomistic simulations with DFT Hamiltonian and the Perdew–Burke–Ernzerhof (PBE) exchange‐correlation functional were performed to investigate the electronic structure of the armed structures [65]. Scalar‐relativistic Goedecker–Teter–Hutter (GTH) pseudopotentials were employed for all atoms, replacing the core electrons, while the valence electrons were described by a double‐𝜁 basis set plus polarization functions [66] as implemented in the CP2K 6.1 package [67]. The auxiliary plane–wave cutoff was set to 400 Ry. All calculations were performed in vacuum within non‐periodic cubic simulation boxes of 65 Å on the side for the long, medium, and short‐armed CsPbBr_3_ NCs, respectively. Structural relaxations were carried out until the maximum residual force on atoms was below 4.5 × 10^−4^ Ha Bohr^−1^. Additionally, the delocalization of frontier molecular orbitals was quantified by computing the PR and IPR, defined as:

$$PR_{i}=\frac{{\left(\sum_{\alpha }{\left|P_{\alpha ,i}\right|}^{2}\right)}^{2}}{\sum_{\alpha }{\left|P_{\alpha ,i}\right|}^{4}}$$

and,

$IPR_{i}=\frac{1}{PR_{i}}$where *P*_α,*i*_ corresponds to the weight of the molecular orbital *i* on a given atom α expanded on an atomic orbital basis.

IPR values significantly larger than zero reveal the presence of localized trap states in the electronic structure, which we find at the bottom of the CB in all armed structures. Following our previous work [68], we used an IPR threshold of 0.03 to filter out these trap states and identify the actual band edges.

### 4.4 Optical Properties Measurement

#### 4.4.1 Measurement of Absorption, Photoluminescence (PL) and Photoluminescence Quantum Yield (PLQY)

Absorption of CsPbBr_3_ NCs solution was measured using an Agilent Cary 60 UV–vis spectrometer. The optical bandgap was estimated using the Tauc plot method. PL spectra were recorded along with PLQY measurement using a Horiba Fluorolog 3.22 spectrofluorimeter with an F‐3029 integrating sphere accessory fitted with a cuvette holder to accommodate NCs solutions for quantum yield quantification. A 450‐Watt xenon lamp was used as an excitation source (365 nm).

#### 4.4.2 Time‐Resolved PL

Time‐resolved PL decay measurements were performed using a confocal fluorescence microscope, Leica TCS SP8 equipped with a 40X (1.10 NA) water immersion objective lens (HC PL IRAPO, Leica Microsystems). Excitation was provided at 488 nm with a pulse repetition rate of 3.1 MHz, generated by a supercontinuum laser. Emission was detected in the 500–600 nm spectral range using a hybrid detector optimized for high sensitivity and an enhanced signal‐to‐noise (S/N) ratio. The PL decay dynamics of individual particles were analyzed by fitting the decay profiles with a bi‐exponential function, from which the fitting parameters and intensity‐weighted average lifetimes were extracted. This analysis was conducted on an average of 20 individual particles of each type to assess heterogeneity and variations in lifetime distribution across different particles.

#### 4.4.3 PL Blinking Measurements

Single particle PL blinking measurements were performed in a standard homebuilt widefield fluorescence microscope integrating an Olympus IX microscope body and 60X (0.9 NA) dry objective. Samples were excited with a 488 nm continuous wave laser, and a PL movie was recorded with a CMOS camera with a 10 ms exposure time. PL blinking traces are extracted and analyzed using home‐made MATLAB‐based software. Power spectral density estimation of each blinking time trace was performed by Fourier transformation of the intensity autocorrelation function according to the Wiener–Khinchin theorem. Details of the methodologies can be found in [36]. The excitation power‐dependent measurements were performed using the same setup by varying the power from 0.1 to 41 W/cm^2^. PL intensity at each power was calculated by integrating the total counts of each blinking trace corresponding to individual NCs.

## Funding

I.S acknowledges the support of the Research Foundation‐Flanders SB‐FWO fellowship 1SHA024N, S.S. acknowledges the support of Marie Sklodowska‐Curie postdoctoral fellowship No. 101151427, SPS_Nano from the European Union's Horizon Europe program, J.Z. acknowledges FWO grant 12AA526N, R.G. acknowledges support from a FWO fellowship under award 12A1V25N, and B.V.H. acknowledges FWO grant 1SACB26N. A.D.B., S.V.A., and S.B. acknowledge the financial support from FWO (Grant G0A7723N). S.A. acknowledges funding from VLAIO HBC.2022.0185 Baekeland mandate. J.H. acknowledges financial support from FWO (Grant Nos. G098319N, G0F2322N, S002019N, VS06523N, and G0AHQ25N), from the Flemish government through long‐term structural funding Methusalem (CASAS2, Meth/15/04), and the MPIP as an MPI fellow. E.D. acknowledges funding from the KU Leuven Internal Funds (grant numbers C14/23/090 and CELSA/23/018), FWO, grant number G0AHQ25N, and the European Union (ERC Starting Grant, 101117274 X‐PECT). However, the views and opinions expressed are those of the authors only and do not necessarily reflect those of the European Union or European Research Council. Neither the European Union nor the granting authority can be held responsible for them.

## Conflicts of Interest

The authors declare no conflicts of interest.

## Supporting information




**Supporting File 1**: adma72815‐sup‐0001‐SuppMat.docx.

**Supporting File 2**: adma72815‐sup‐0002‐MovieS1.gif.

**Supporting File 3**: adma72815‐sup‐0003‐MovieS2.gif.

**Supporting File 4**: adma72815‐sup‐0004‐MovieS3.avi.

**Supporting File 5**: adma72815‐sup‐0005‐MovieS4.avi.

**Supporting File 6**: adma72815‐sup‐0006‐MovieS5.avi.

## Data Availability

The data that support the findings of this study are available from the corresponding author upon reasonable request.
