# Supplementary material for: Arm‐Length‐Controlled CsPbBr3 Nanocrystals for Tunable Optical and Assembly Behavior
Source: Adv Mater. 2026 Mar 24;38(23):e19211. doi: 10.1002/adma.202519211 (PMC13103628; doi:10.1002/adma.202519211)
Supplement: Supplementary file 1 — Supporting File 1: adma72815‐sup‐0001‐SuppMat.docx. [file ADMA-38-e19211-s004.docx]

Supporting Information

**Arm-Length Controlled CsPbBr_3_ Nanocrystals for Tunable Optical and Assembly Behavior**

*Irina Skvortsova, Sudipta Seth, Juliette Zito, Robin Girod, Bob Van Hout, Annick De Backer, Tom Stoops, Sergey Abakumov, Evgenii Vlasov, Tejmani Behera, Sandra Van Aert, Elke Debroye*, Johan Hofkens*, Sara Bals**

I. Skvortsova, J. Zito, R. Girod, A. De Backer, T. Stoops, E. Vlasov, S. Van Aert, S. Bals

Electron Microscopy for Materials Science (EMAT) & NANOlight Center of Excellence

University of Antwerp

2020 Antwerp, Belgium

E-mail: [sara.bals@uantwerpen.be](mailto:sara.bals@uantwerpen.be)

S. Seth, B. Van Hout, S. Abakumov, T. Behera, E. Debroye, J. Hofkens

Department of Chemistry

KU Leuven

3001 Leuven, Belgium

E-mail: [elke.debroye@kuleuven.be](mailto:elke.debroye@kuleuven.be), johan.hofkens@kuleuven.be

J. Hofkens

Max Planck Institute for Polymer Research

55128 Mainz, Germany

E-mail: johan.hofkens@kuleuven.be

**
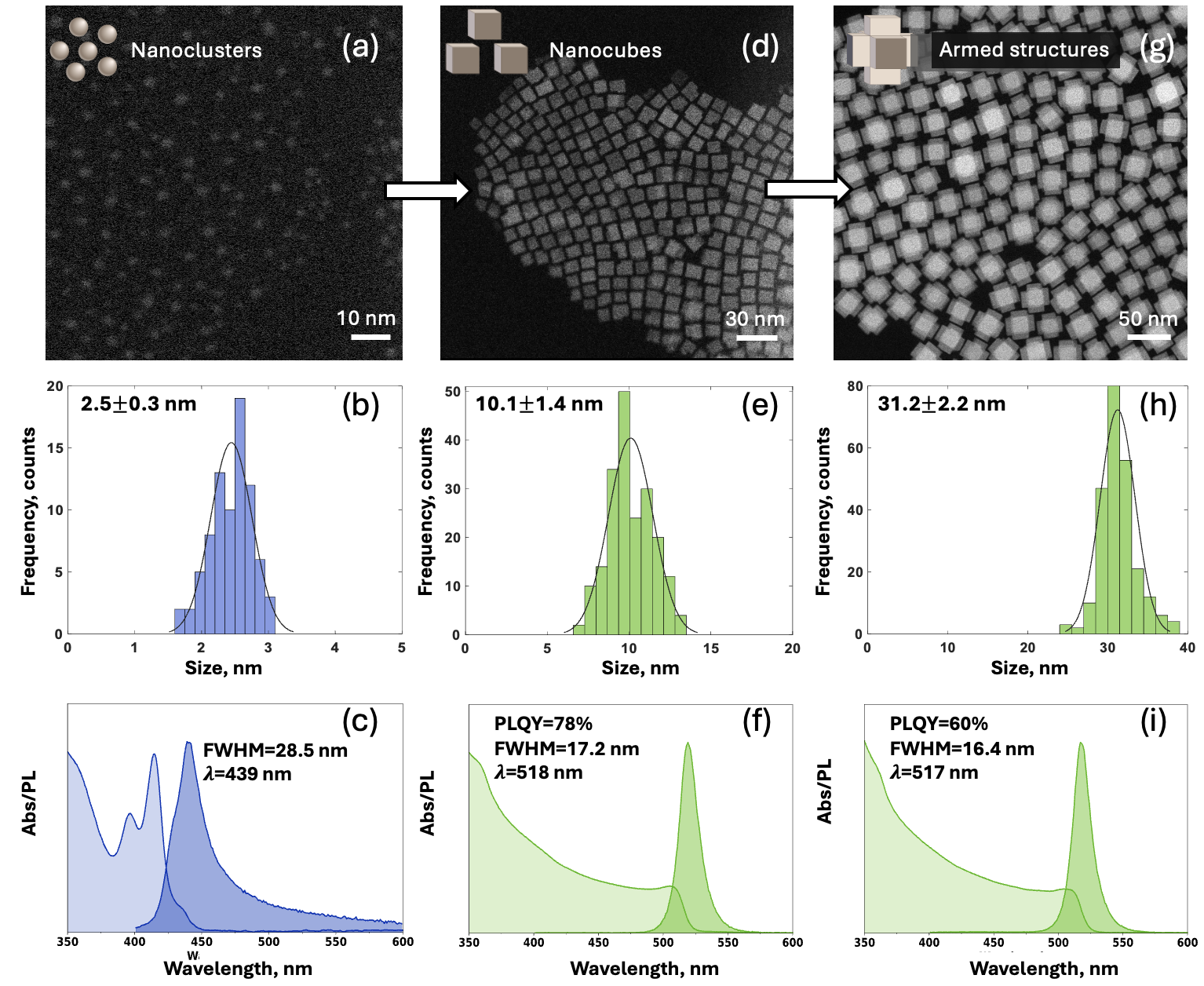
**

**Figure S1.** HAADF-STEM overview images, particle size distribution and absorption/PL data for nanoclusters (seeds) formed at the first step of the reaction (a,b,c), for the nanocubes formed after 0 minutes at the second step of reaction (d,e,f) and for the armed structures formed after 3 minutes at the second step of reaction (g,h,i).


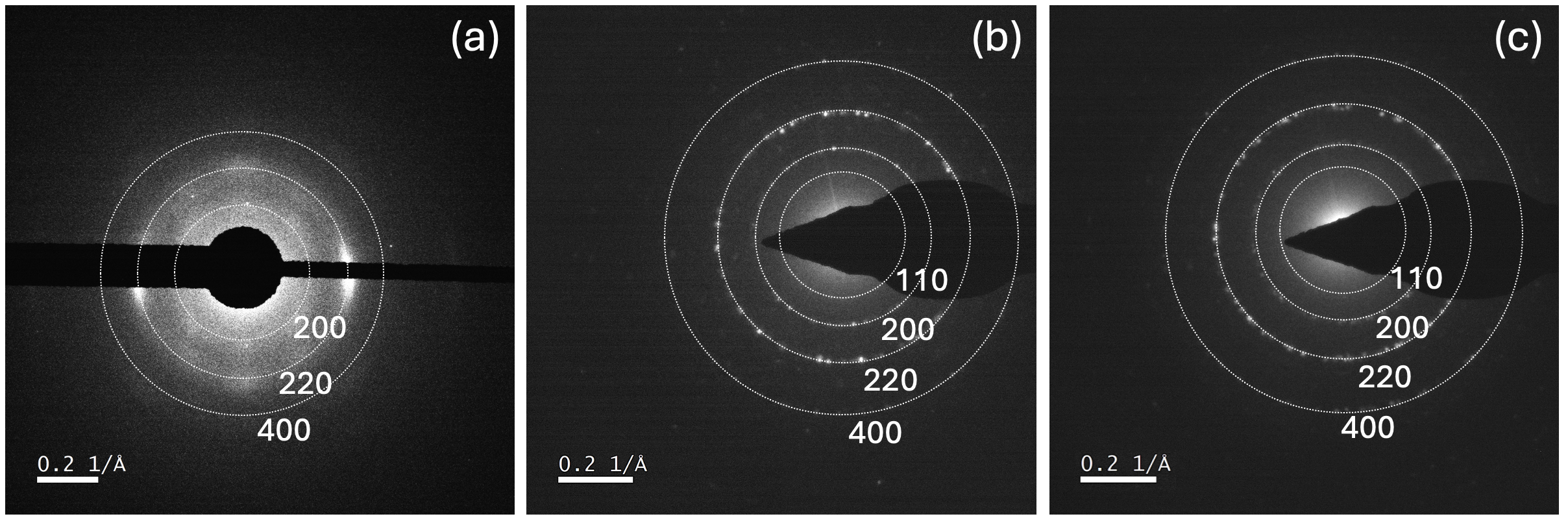


**Figure S2.** SAED patterns for (a) nanoclusters (seeds) formed at the first step of the reaction, (b) nanocubes formed after 0 minutes at the second step of reaction and (c) armed structures formed after 3 minutes at the second step of reaction. Patterns are indexed in *Pbnm* orthorhombic crystal system.

**Table S1.** Experimental particle sizes, arm lengths, core sizes and arm-to-core size ratios for three different armed samples obtained with 0.3 mL of Cs-oleate (long), 0.2 mL of Cs-oleate (middle) and in toluene after 1 day (short).

|  | *Particle size, nm* | *Arm length, nm* | *Core size, nm* | *Arm-to-core ratio* |
| --- | --- | --- | --- | --- |
| Long arm | 40.7$\pm$3.5 | 8.9$\pm$1.4 | 22.9$\pm$4.5 | 0.39$\pm$0.07 |
| Middle arm | 31.2$\pm$2.2 | 5.7$\pm$0.7 | 19.8$\pm$2.6 | 0.29$\pm$0.04 |
| Short arm | 28.1$\pm$1.6 | 3.7$\pm$0.5 | 20.7$\pm$1.9 | 0.18$\pm$0.03 |


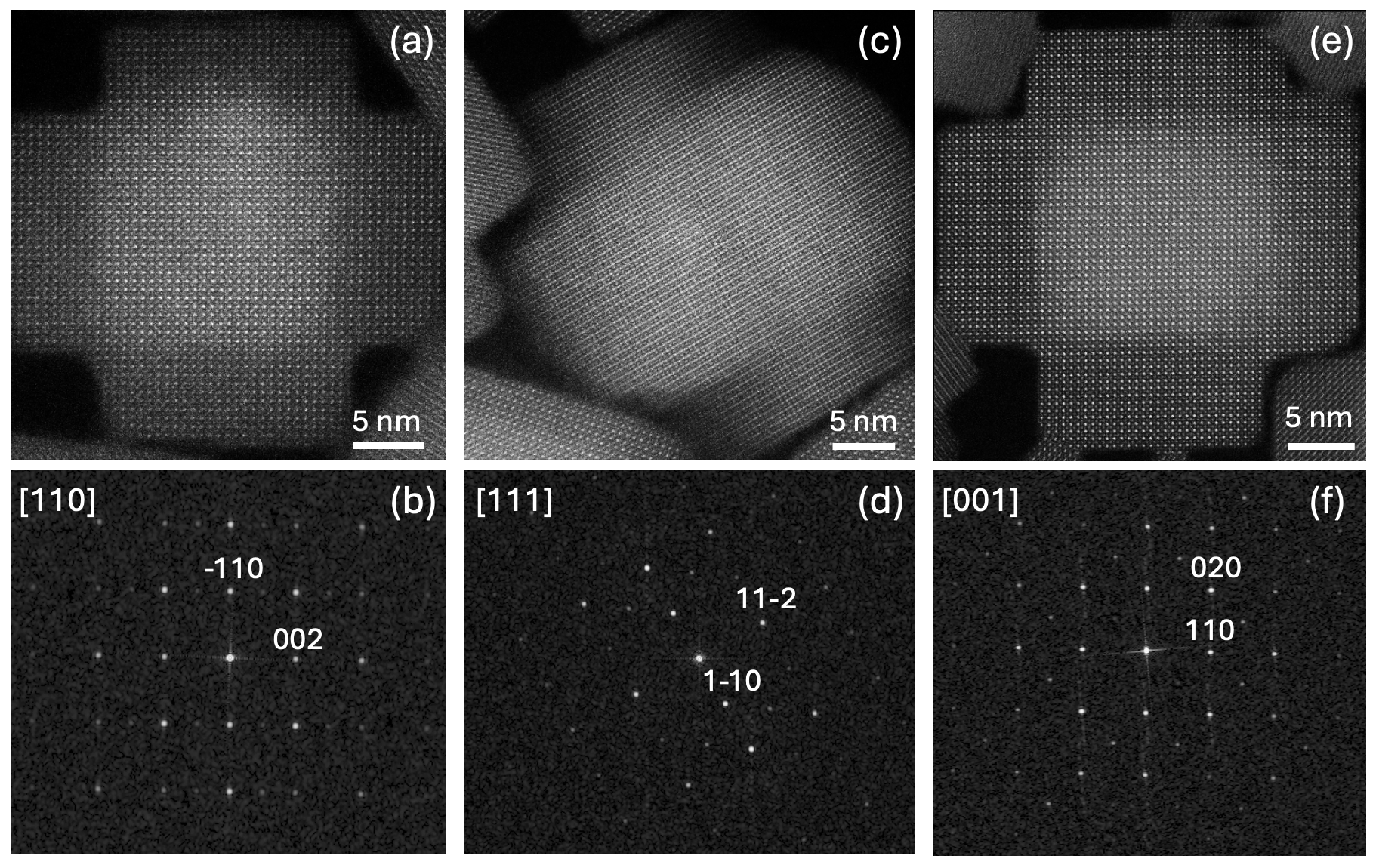


**Figure S3.** High-resolution HAADF-STEM images with the corresponding Fourier Transform (FT) patterns for the armed structures aligned along [110] (a, b), [111] (c, d) and [001] (e, f) crystallographic directions.


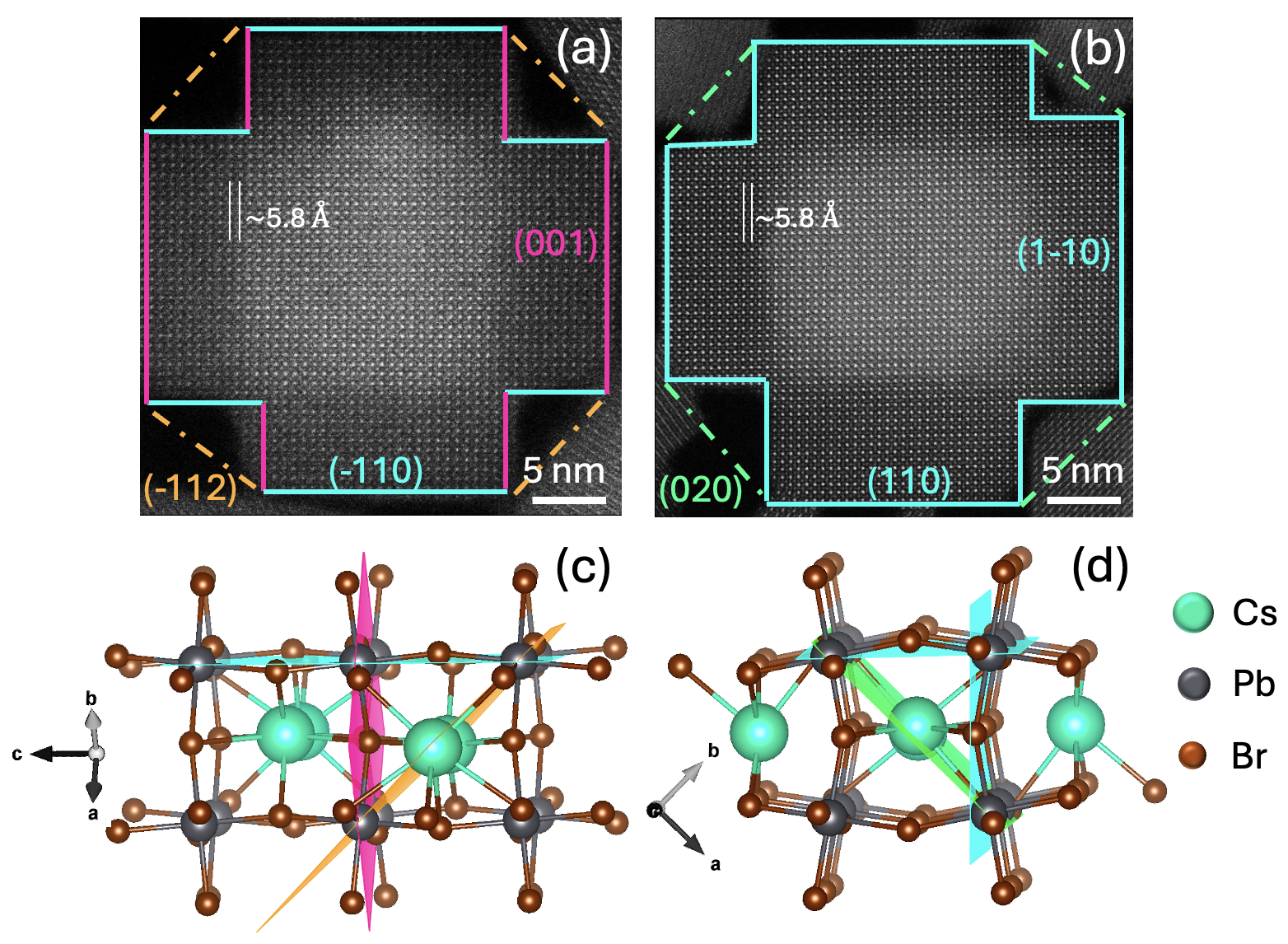


**Figure S4.** High-resolution HAADF-STEM images (a, b) of single CsPbBr_3_ armed structures imaged along the [110] and [001] zone axes (orthorhombic crystal system, *Pbnm*) with corresponding crystal structure models (c, d).


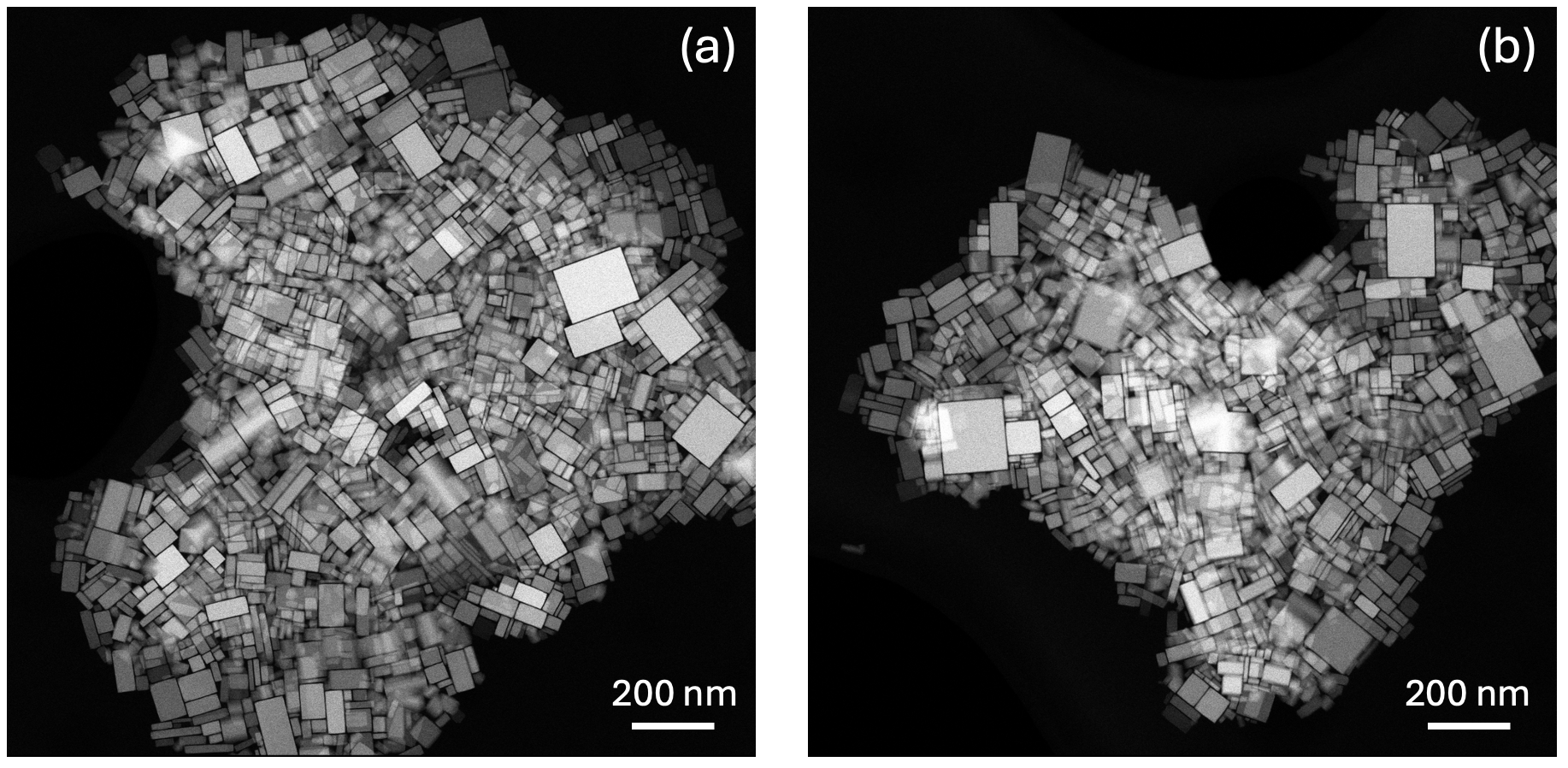


**Figure S5.** HAADF-STEM overview images (a, b) of the NCs prepared when seeds are injected into the ODE with 0.5 ml of oleic acid and 0.5 ml of oleylamine.


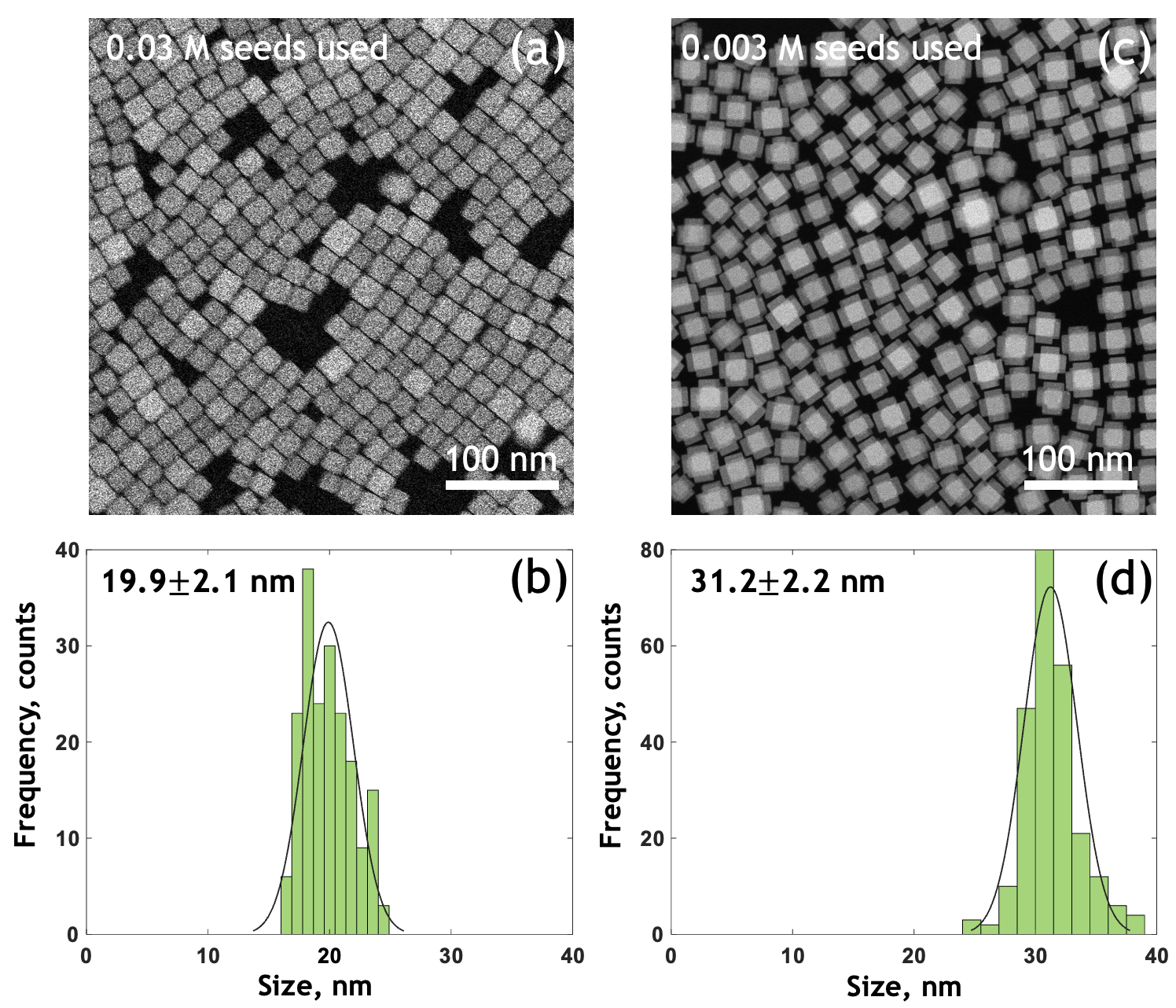


**Figure S6.** HAADF-STEM overview images with the corresponding particle size distributions of the synthesis product in case of 0.03 M (a,b) and 0.003 M (c,d) seeds solution usage for the injection into 3 mL of ODE at 245 ℃.


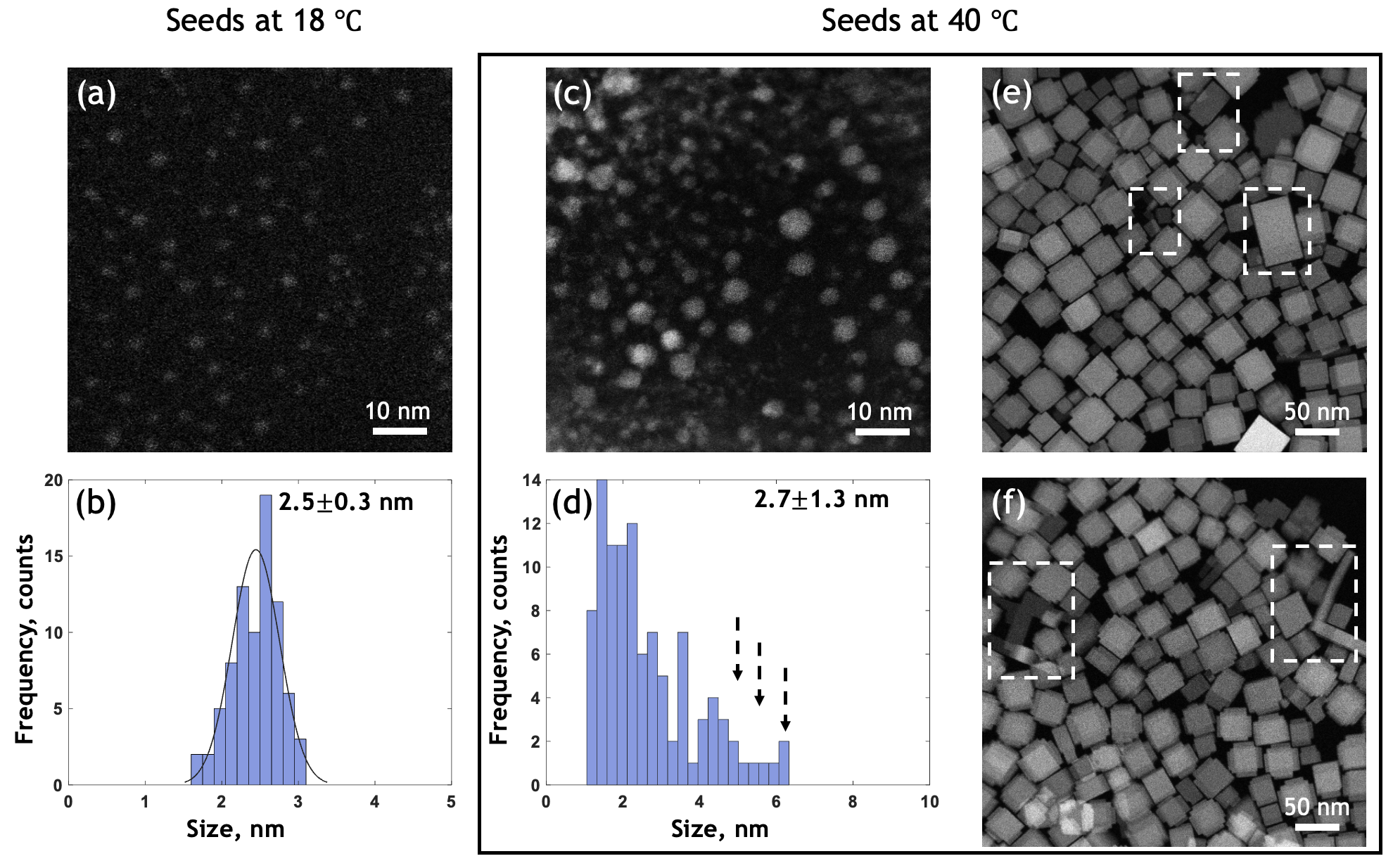


**Figure S7.** HAADF-STEM overview images with the corresponding particle size distributions of the CsPbBr_3_ seeds prepared at 18 ℃ (a,b) and at 40 ℃ (c,d). Black dashed arrows in (d) highlight the presence of agglomerated seeds in a solution. HAADF-STEM overview images of the synthesis product after the injection of the seeds prepared at 40 ℃ into 3 mL of ODE at 245 ℃ (e, f). White dashed rectangles in (e, f) highlight the presence of the random/irregular shapes.

**
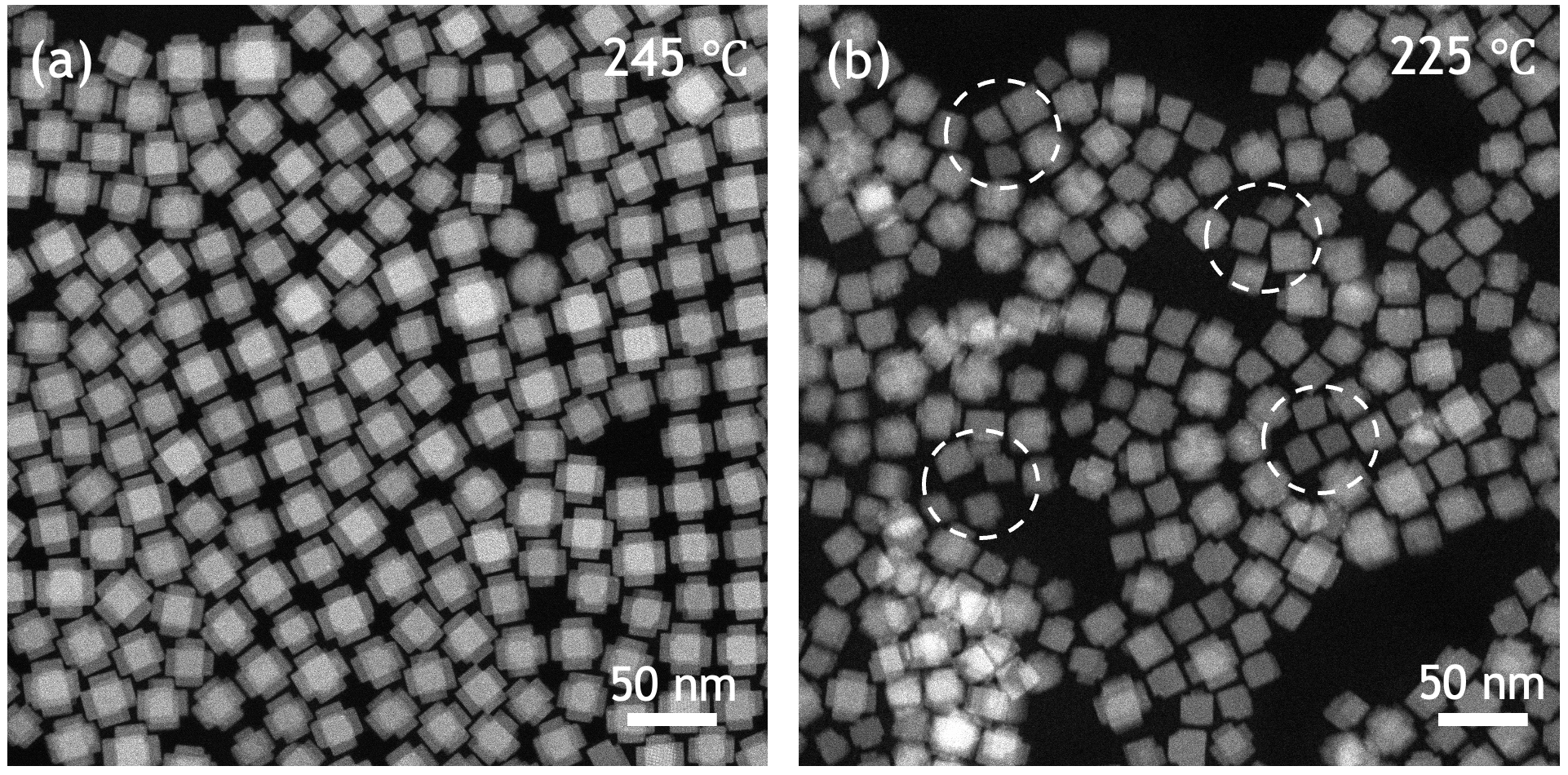
**

**Figure S8.** HAADF-STEM overview images of the CsPbBr_3_ armed structures prepared at 245 ℃ (a) and at 225 ℃ (b). White dashed circles in (b) highlight the presence of the cuboidal morphology.


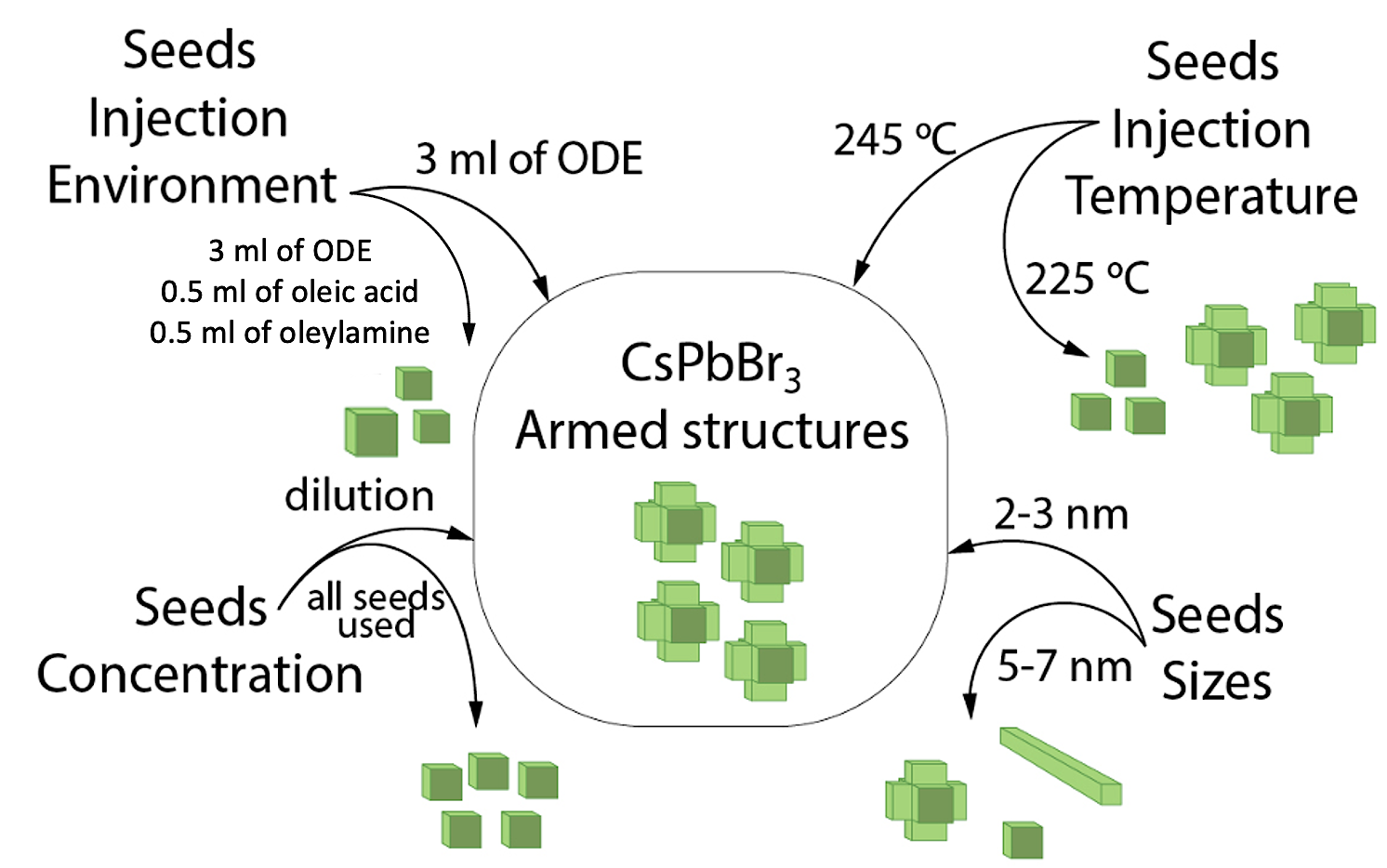


**Figure S9.** Schematic summary of the main synthetic parameters leading to the formation of the CsPbBr_3_ armed morphology.

**
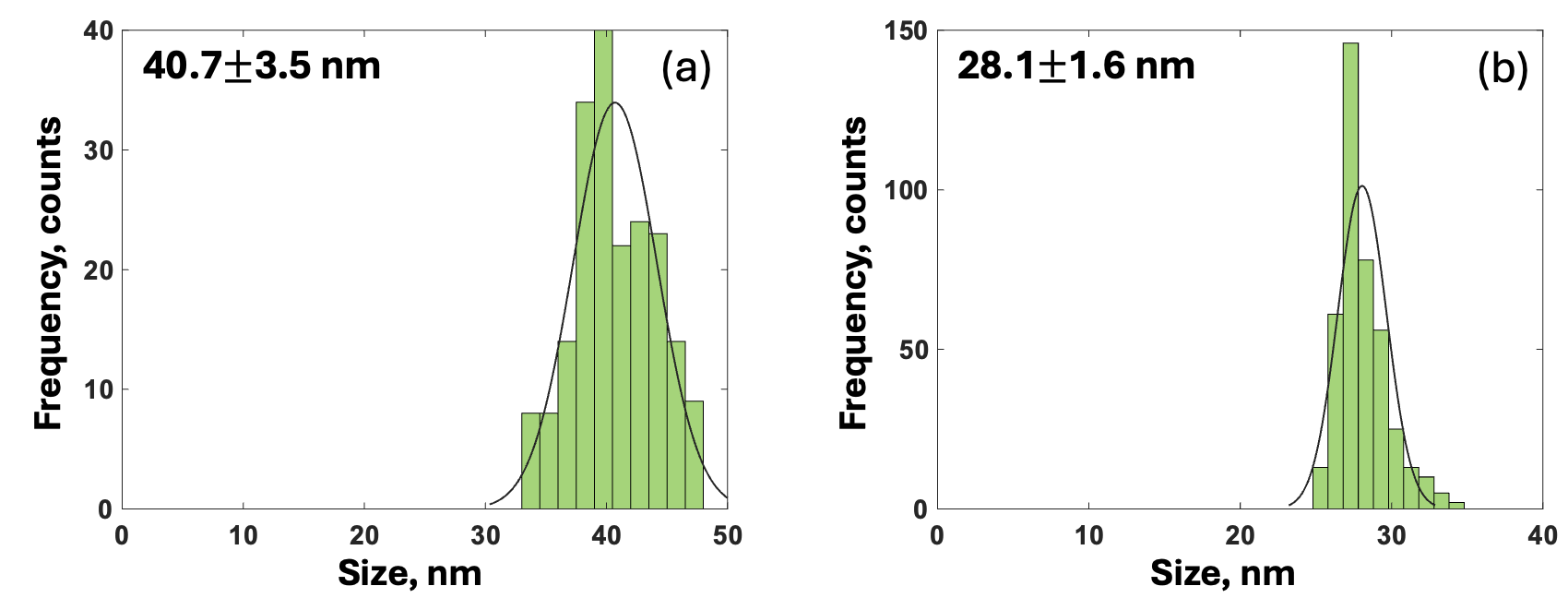
**

**Figure S10.** Particle size distribution for the NCs obtained as a result of the synthesis with 0.3 mL of Cs-oleate (a), i.e. for long-armed structures and for the NCs obtained as a result of 1 day exposure of the armed structures with the middle length (synthesis with 0.2 mL of Cs-oleate) to the air in toluene at 20 ℃ (b), *i.e.* for short-armed structures.

**
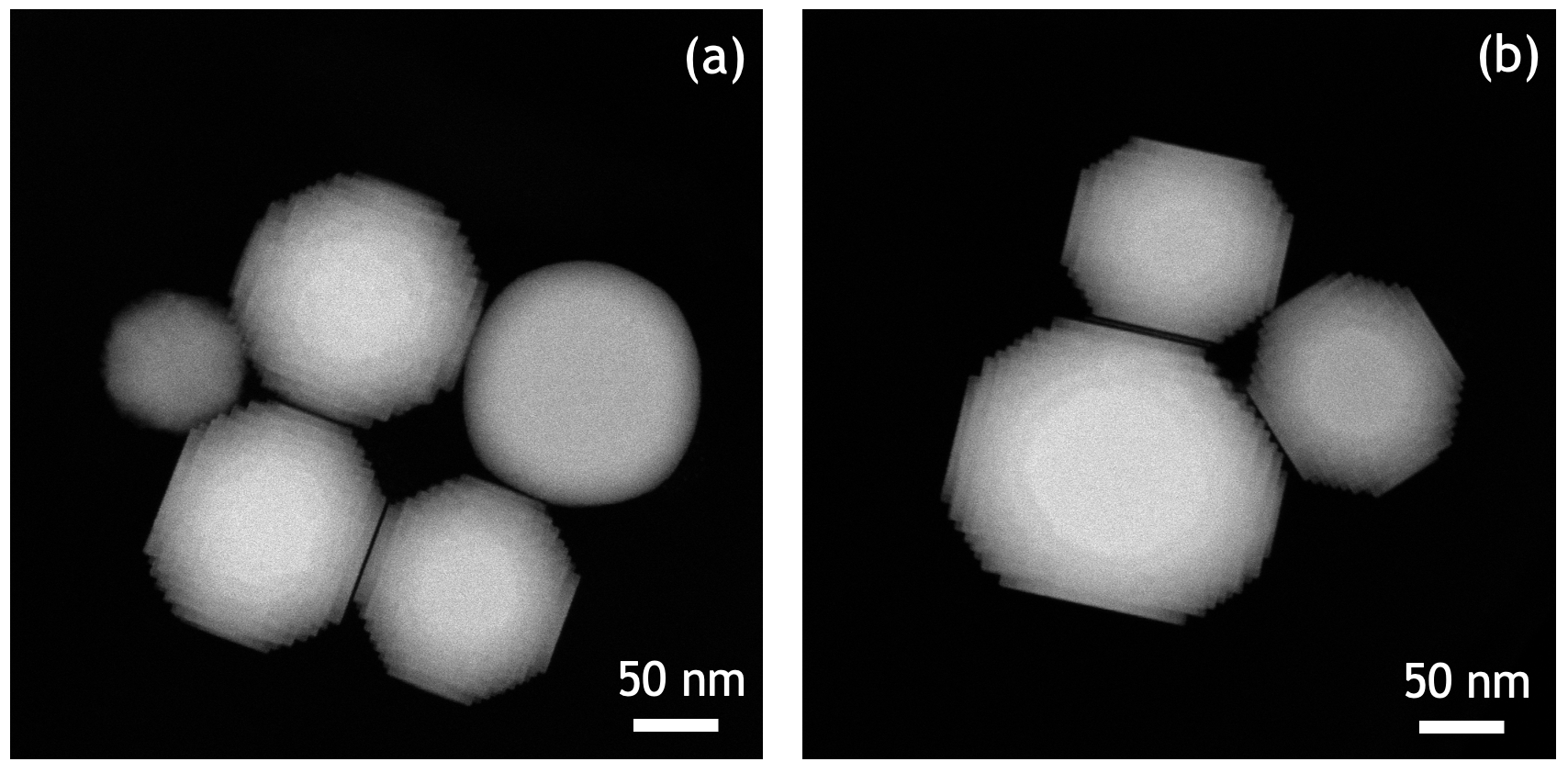
**

**Figure S11.** HAADF-STEM overview images (a, b) of the particles obtained as a result of the synthesis with 0.1 mL of Cs-oleate.

**
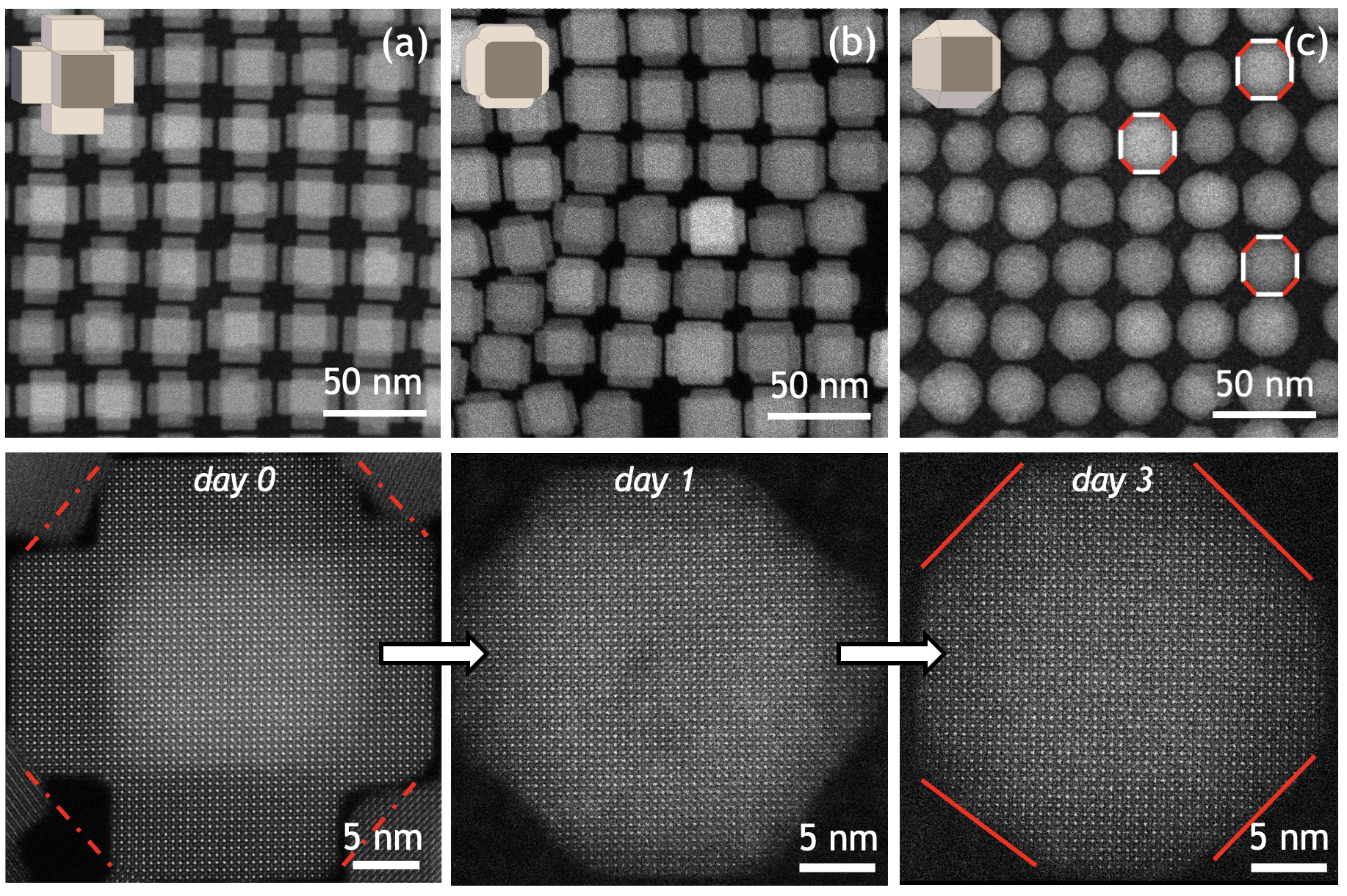
**

**Figure S12.** HAADF-STEM overview as well as high-resolution images following the shape evolution of the armed structures with the middle arm length in time. Initial middle armed structures (a), after 1 day of storage in toluene exposed to the air at 20 ℃ (b), after 3 days of storage in toluene exposed to the air at 20 ℃ (c). The red lines in (a) and (c) highlight {110}_c_ facets which are initially absent and appear after 3 days of storage. This morphological transformation can be attributed to two primary factors: (i) different polarity index of toluene (P=2.4) and hexane (P=0.1), and (ii) relatively high hygroscopicity of toluene (500 mg/L of water) compared to hexane (9.5 mg/L of water).^[1]^ Firstly, since hexane is nonpolar, it has very weak interactions with both the oleic acid and oleylamine ligands. This means that hexane is less likely to disrupt the coordination between the ligands and the surface of the NCs, leading to high morphological stability of the NCs over time. On the other hand, toluene is a slightly polar aprotic solvent, which means it has a moderate ability to interact with polar functional groups, such as the carboxyl group (-COOH) of oleic acid and the amine group (-NH_2_) of oleylamine. When the perovskite NCs are exposed to toluene for some time, the ligands can desorb into toluene, leaving the crystal surface less passivated and making the surface more reactive. This desorption can result in the exposure of more surface defects or unsaturated bonds, which are more susceptible to dissolution/recrystallization or reorganization. In this manner the formation of the 26-faceted rhombicuboctahedron (**Figure S13c,d**) over time is driven. Additionally, toluene can form π–hydrogen bonds with water molecules,^[2]^ which enhances its capacity to absorb atmospheric moisture. Adsorbed water molecules can interact with surface-exposed Pb²⁺ and Br⁻ ions, promoting halide or metal loss, and further destabilizing the crystal structure. This increased surface reactivity can ultimately lead to the thermodynamically favored rhombicuboctahedral morphology (**Figure S12, Figure S13c,d**). The mechanisms of the transition can be either dissolution-reprecipitation or surface reconstruction (atoms diffusion). Since the particle maintains single crystallinity and initial orientation, we assume that surface reconstruction is more likely at the given size scale (few nm).


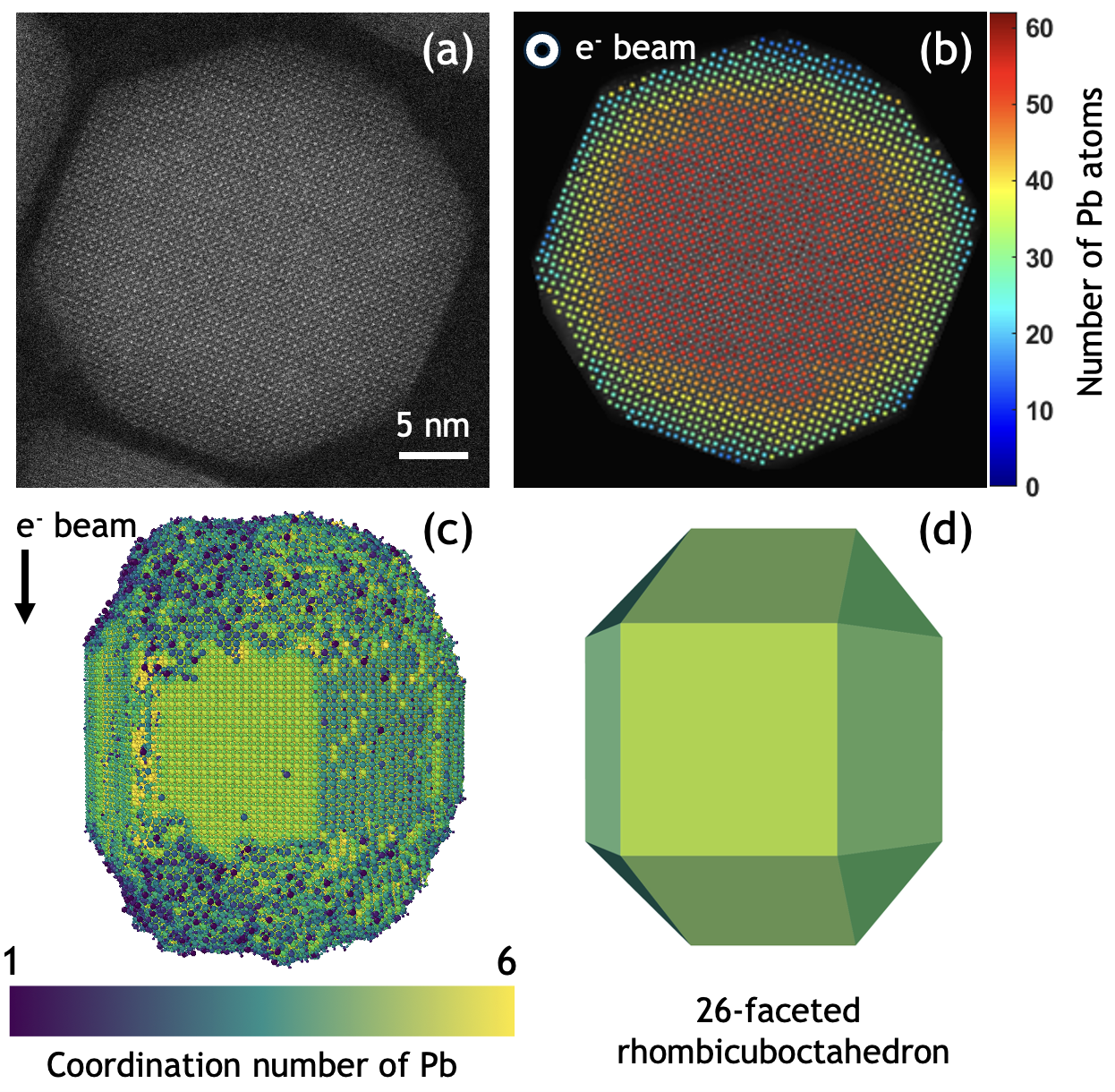


**Figure S13.** High-resolution HAADF-STEM image of the NC aligned along [110] direction obtained as a result of 3 days storage of the middle-armed structure in toluene exposed to the air at 20 ℃ (a). The color-coded map showing the distribution of the number of Pb atoms in every column (b). Experimental atomic 3D shape of the NC with Pb coordination analysis performed for each column for clear representation of the facets (c). Schematic model of the 26-faceted rhombicuboctahedron (d) in the same orientation as the experimental atomic shape of the NC shown in (c).

**Table S2.** Absorption and PL data analysis for the NCs with different arm lengths.

|  | Excitonic peak, eV | Band gap, eV | PL peak position, nm | FWHM of PL peak, nm | PLQY, % |
| --- | --- | --- | --- | --- | --- |
| Long arm | 507 | 2.38 | 517 | 17.3 | 69 |
| Middle arm | 508 | 2.38 | 517 | 16.4 | 60 |
| Short arm | 510 | 2.37 | 518 | 16.9 | 40 |


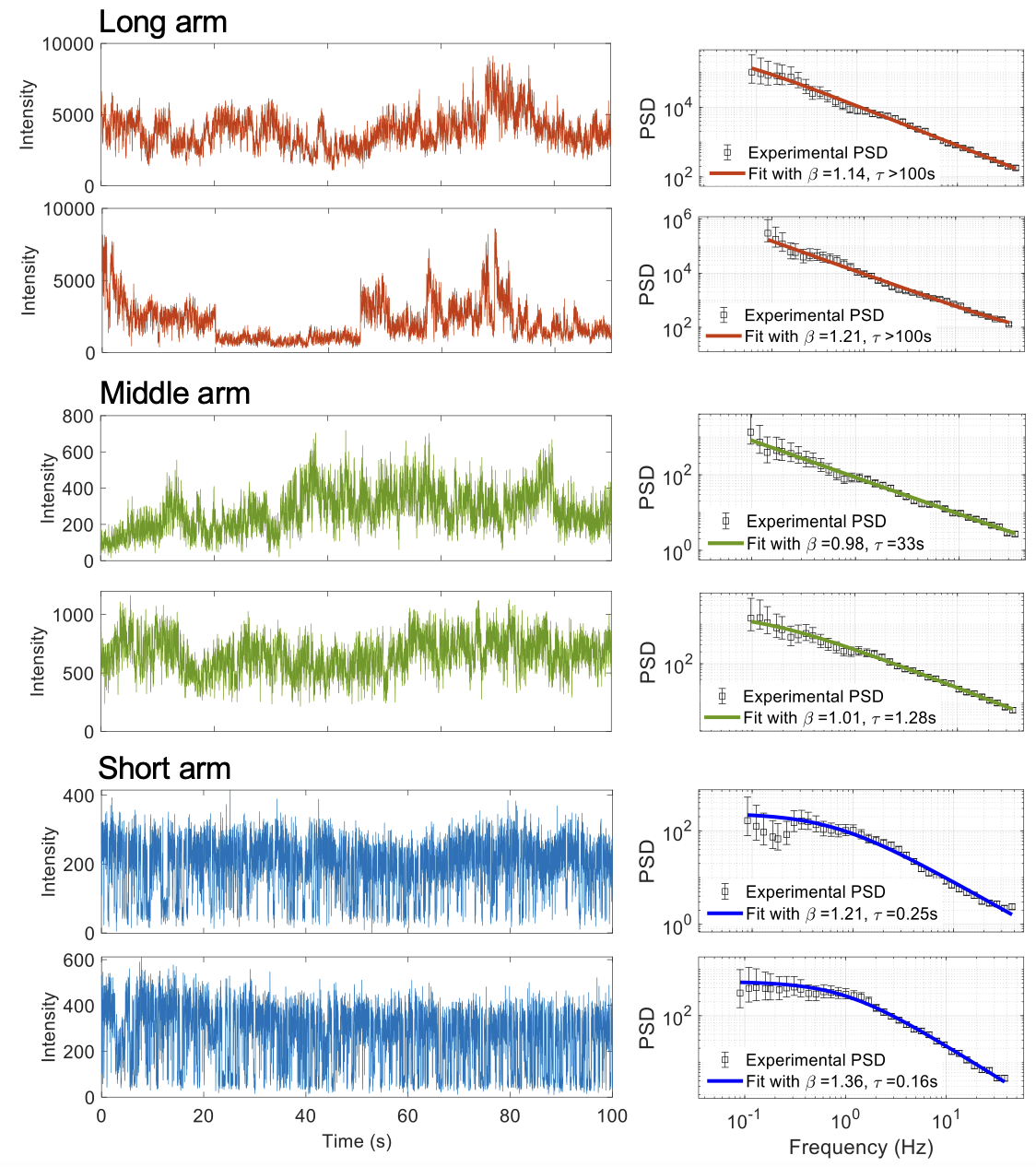
**Figure S14**. Additional examples of PL blinking and corresponding power spectral density of NCs with different arm lengths.


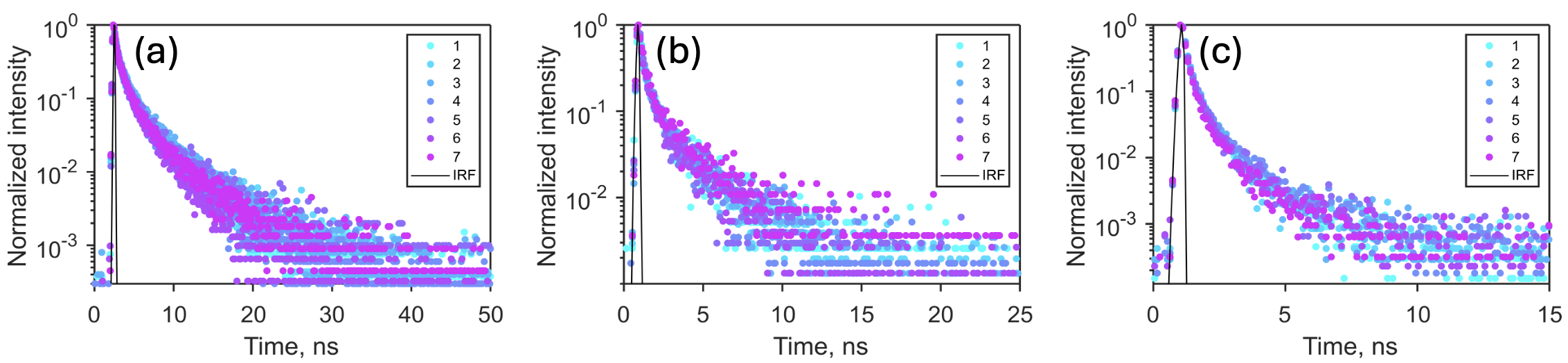


**Figure S15**. Time-resolved photoluminescence decay profiles of several individual armed NCs, plotted in different colors and shapes, with long (a), middle (b) and short (c) arms. IRF corresponds to Instrument Response Function.

**Table S3**. Time-resolved fluorescence parameters of CsPbBr_3_ NCs with different arm lengths achieved experimentally.

|  | t_1_, ns | $\alpha$_1_ | t_2_, ns | $\alpha$_2_ | t_avg_, ns |
| --- | --- | --- | --- | --- | --- |
| Long arm | 0.98$\pm$0.09 | 0.58$\pm$0.04 | 4.89$\pm$0.57 | 0.42$\pm$0.04 | 2.62$\pm$0.32 |
| Middle arm | 0.49$\pm$0.10 | 0.58$\pm$0.33 | 3.39$\pm$0.69 | 0.42$\pm$0.03 | 1.71$\pm$0.28 |
| Short arm | 0.22$\pm$0.08 | 0.68$\pm$0.08 | 1.21$\pm$0.71 | 0.31$\pm$0.08 | 0.60$\pm$0.19 |

**
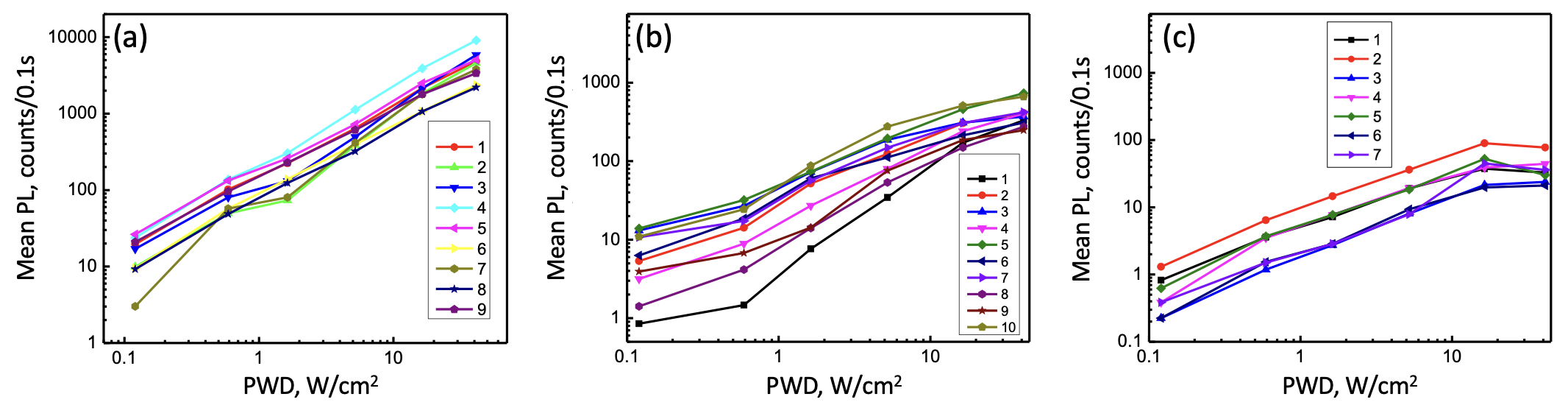
**

**Figure S16.** Excitation power density dependent PL of (a) long-, (b) middle- and (c) short-armed structures.


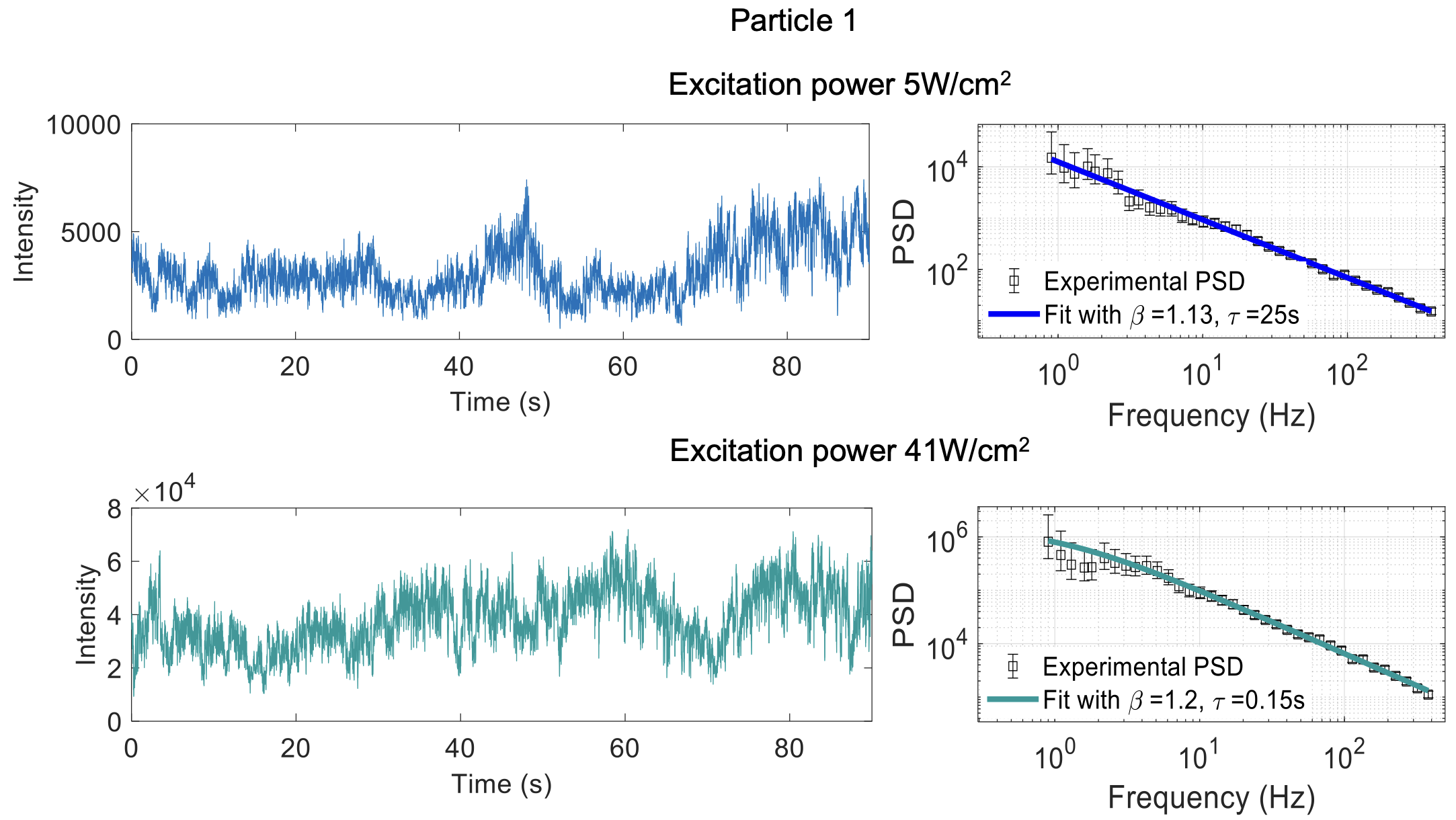


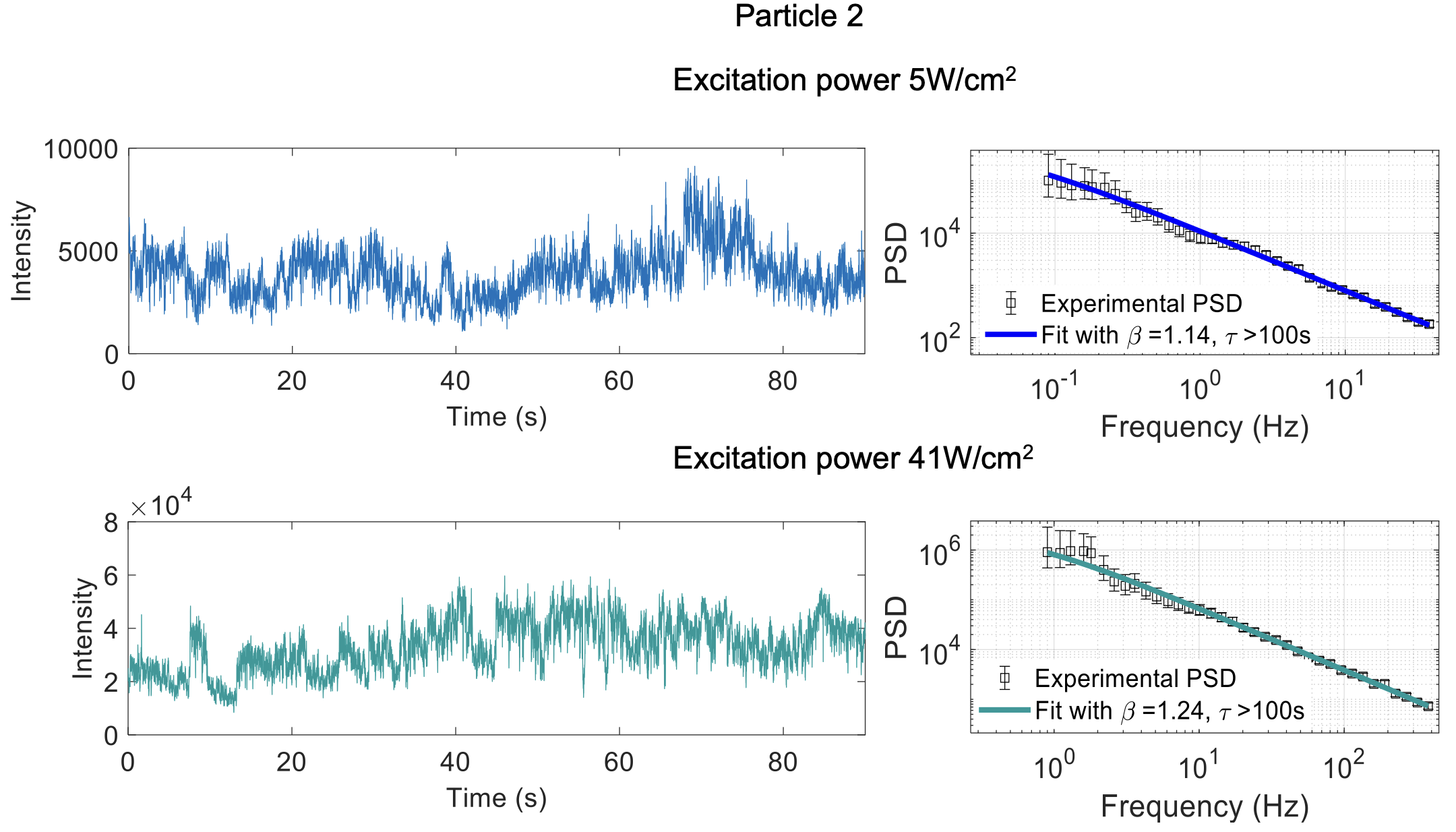


**Figure S17.** Examples of PL blinking and corresponding power spectral density of NCs with long arm length at different excitation power densities.

**
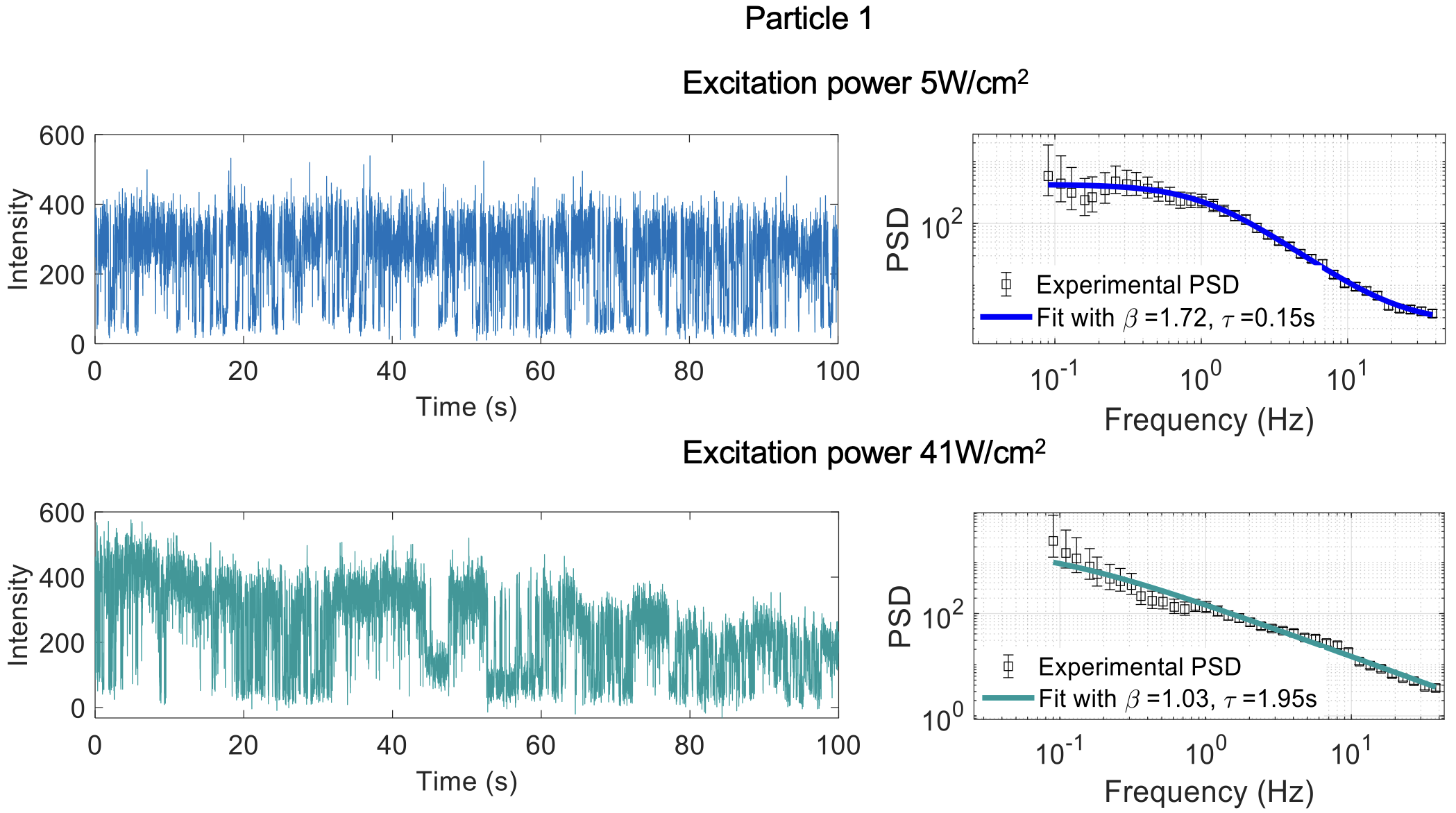
**

**
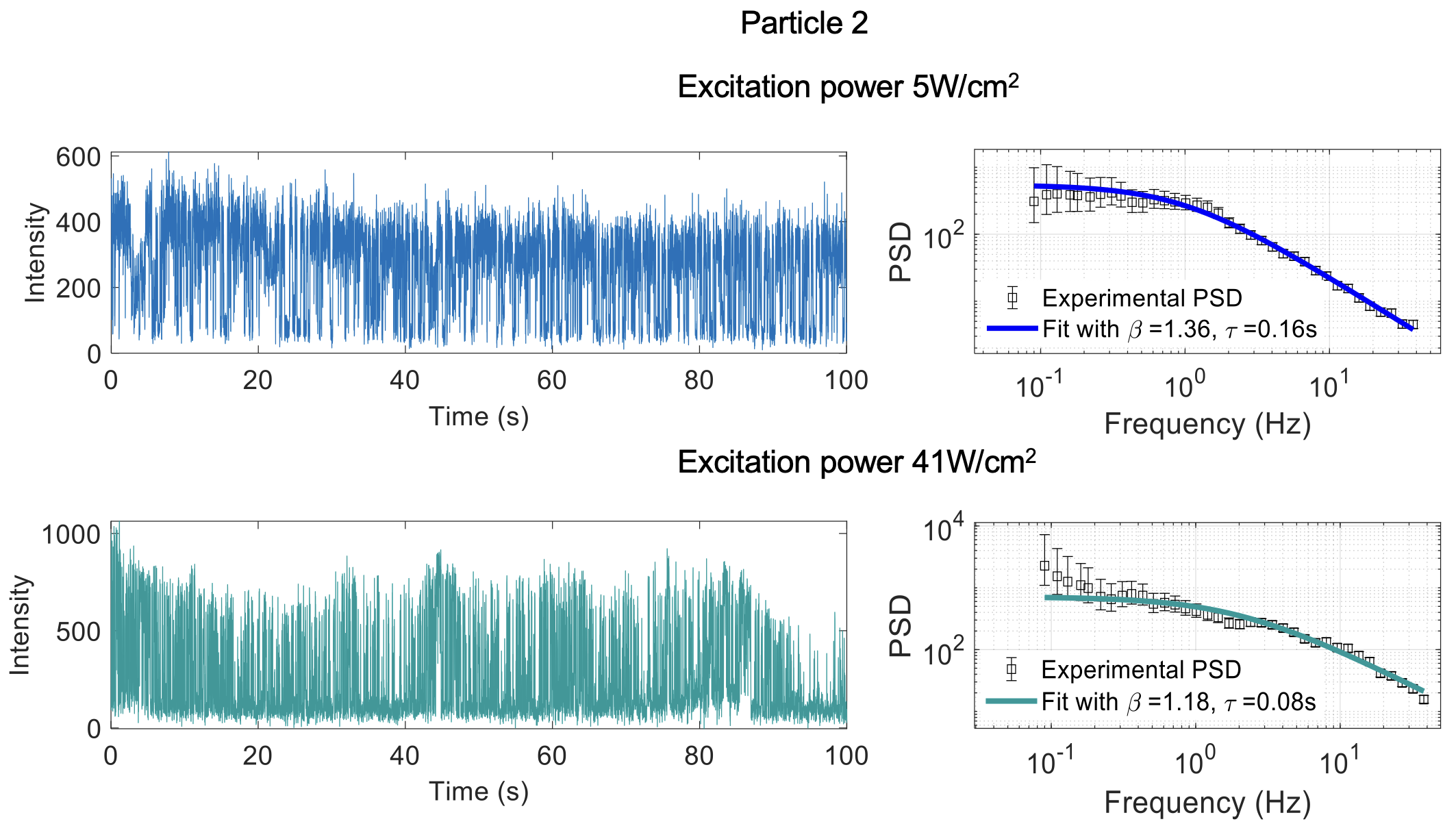
**

**Figure S18.** Examples of PL blinking and corresponding power spectral density of NCs with short arm length at different excitation power densities.

**
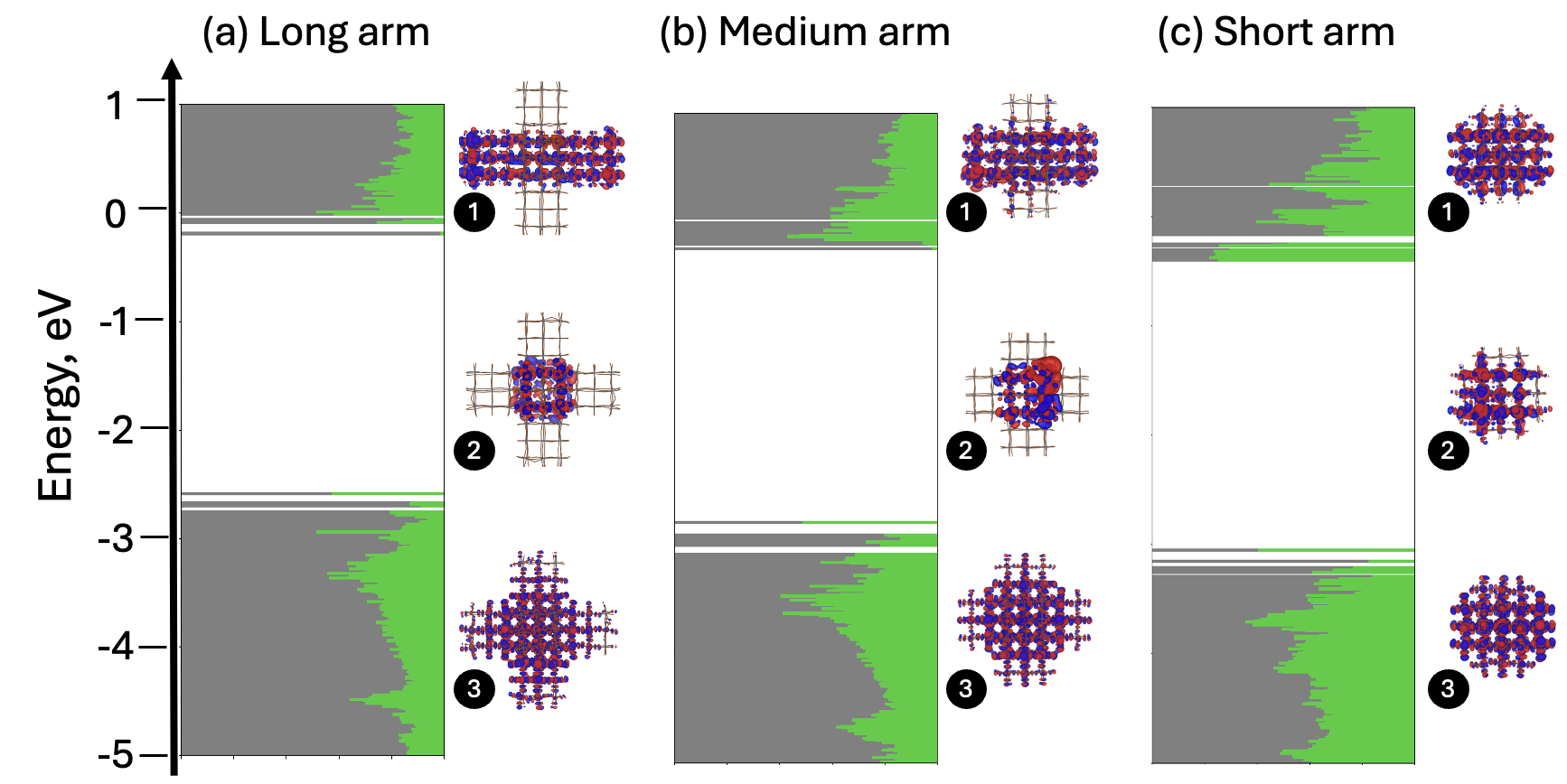
**

**Figure S19.** The electronic structure of (a) long-, (b) medium-_,_ and (c) short-armed CsPbBr_3_ NCs computed at the DFT/PBE level of theory. For each molecular orbital (MO), the length of the different line sections represents the fractional contribution from the arm (gray) and core (green) domains. Isosurfaces of the most relevant molecular orbitals are additionally reported with a counter value of 0.02 e Bohr^−3^, highlighting positive and negative parts in red and blue, respectively. MO1 and MO3 illustrate mixed arm-core contributions at both the CB and VB edges featuring increased delocalization as the arm length increases; MO2 highlight the presence of trap states localized at the surface below the CB. We would like to point out that idealized, fully passivated DFT models, were employed. However, in real samples the long-, medium-, and short-armed structures are produced through different synthetic routes, which inevitably result in different surface chemistry. These differences are not captured by the DFT models and might also play a role in the optical properties. Moreover, the reduced dimensions of the models compared to the experiments result in overestimated arm-to-core ratios of 1.00, 0.67 and 0.33 for the long, medium and short arm cases, which are chosen only to clearly demonstrate the trend of delocalization with the arm length.

**
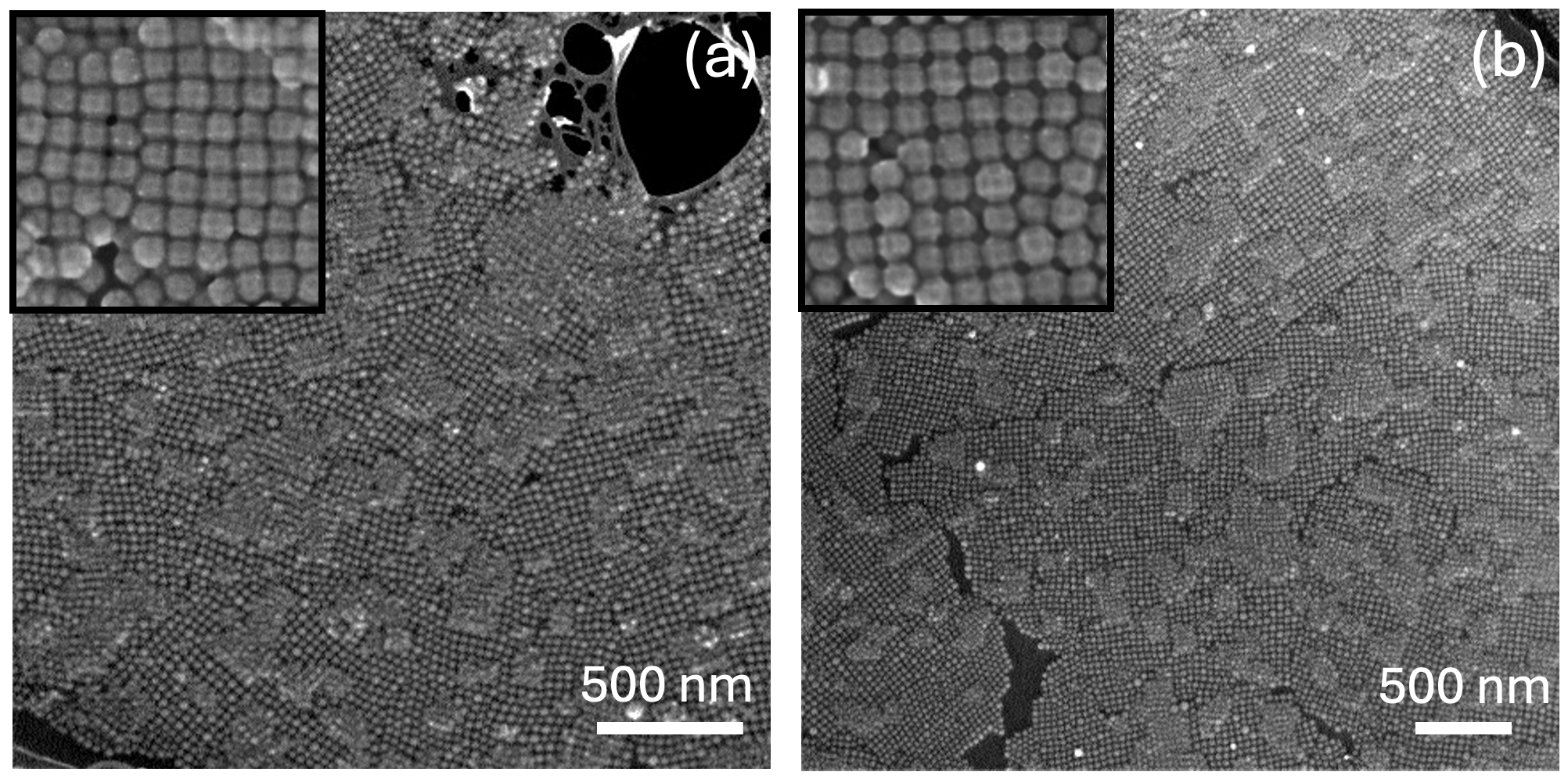
Figure S20.** SEEBIC overview images (a, b) of the assemblies composed of the middle-armed structures with zoomed-in insets.


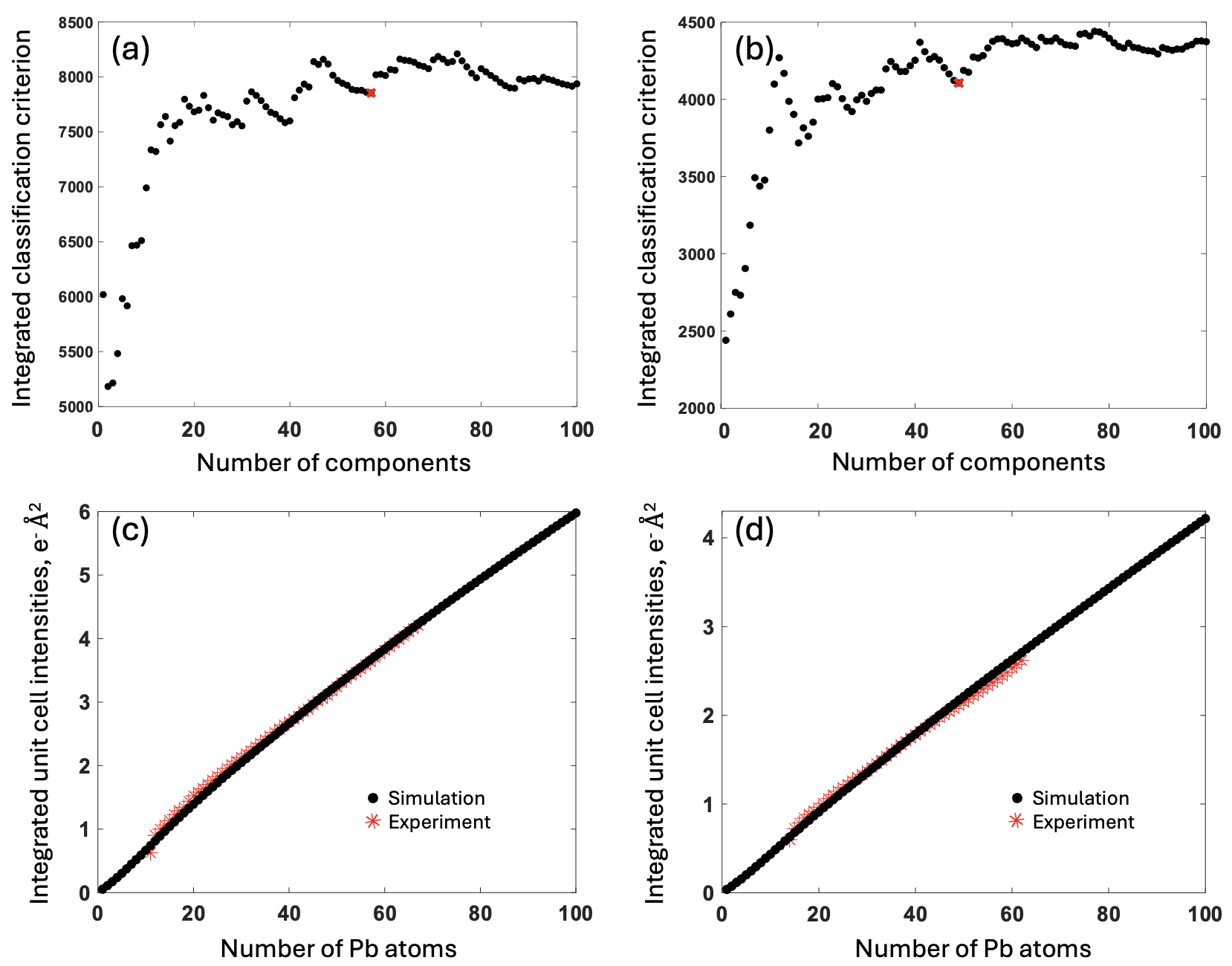


**Figure S21.** Integrated classification criterion as a function of number of components (a,b) and integrated unit cell intensity dependence on the number of atoms in a column - derived from the simulated and experimental data (c,d) for armed NC shown in **Figure 1e** and 26-faceted rhombicuboctahedron shown in **Figure S12b** respectively. Red cross in (a,b) highlight selected number of components to ensure matching between experiment and simulations (c,d).

*Calculation of the facets*

In order to transform planes from orthorhombic ($a_{o}=$8.10 $Å$, $b_{o}=$8.45$Å$ and $c_{o}=$ 11.88 $Å$) into cubic lattice ($a_{c}=b_{c}=c_{c}=$ 5.95 $Å$), we use standard geometry settings which describe the relationship between pseudocubic and orthorhombic basis vectors:

$$a_{o}\sim a_{c}+b_{c}$$

$$b_{o}\sim-a_{c}+b_{c}$$

$$c_{o}\sim2c_{c}$$

Hence, redefined orthorhombic unit cell in cubic coordinates:

$$a_{o}=\left[ 110 \right]_{c}$$

$$b_{o}=\left[ -110 \right]_{c}$$

$$c_{o}=\left[ 002 \right]_{c}$$

Using these basis vectors of the orthorhombic unit cell in cubic coordinates, we can derive the change-of-basis matrix P, reflecting the real space directions:

$P=$ $[a_{o} b_{o} c_{o}]$ =$\left[ \begin{matrix} 1 & -1 & 0 \\ 1 & 1 & 0 \\ 0 & 0 & 2 \end{matrix} \right]$

Planes (Miller indices) correspond to the reciprocal space, so their transformation follows the inverse transpose of the direct space transformation following the general formula:

$\left( hkl \right)_{c}=\left( hkl \right)_{o}*Q$, where $Q=P^{-1}$

Therefore, to convert (-112)_o_ plane into the cubic system:

$\left( -112 \right)_{o}*Q= \left( -112 \right)_{o}*\left[ \begin{matrix} 1/2 & 1/2 & 0 \\ -1/2 & 1/2 & 0 \\ 0 & 0 & 1/2 \end{matrix} \right]$=$\left( -101 \right)_{c}$

And to convert (020)_o_ plane into the cubic system:

$\left( 020 \right)_{o}*Q= (020)*\left[ \begin{matrix} 1/2 & 1/2 & 0 \\ -1/2 & 1/2 & 0 \\ 0 & 0 & 1/2 \end{matrix} \right]$ = $\left( -110 \right)_{c}$

*Calculation of the packing densities*

We analyze the packing of CsPbBr_3_ NCs with arms of length *H* and central core of side *L* into monolayers and trilayers as a function of the shape parameter *x* defined as:

$$x=\frac{H}{L}$$

within the hard particle approximation.

**Monolayers.** The 2D packing fraction (area fraction of the unit cell occupied by the inorganic NC) is denoted as $\eta$*_2D,i_(x)* where *i* is the arrangement of the CsPbBr_3_ NCs in the monolayer.

The first arrangement is described by a square primitive (*sp*) lattice with plane group *p4mm* containing one NC in Wyckoff position 1a. The lattice constant is $a=L+2H$, hence:

$$\eta_{2D,sp}= \frac{L^{2}+4LH}{{(L+2H)}^{2}}, \eta_{2D, sp}(x)= \frac{1+4x}{\left( 1+2x \right)^{2}}$$

The second arrangement is described by a centred rectangular (*cr*) lattice with plane group *cmm* containing two NCs in Wyckoff positions 1a and 1b. The lattice constants are $a=2(L+H)$ and $b=2L$, hence:

$$\eta_{2D,cr}= \frac{{2(L}^{2}+4LH)}{4L(L+H)}, \eta_{2D, cr}(x)= \frac{1+4x}{2(1+x)}$$

We notice that the presence of ligands on the surface of the NCs can increase the lattice constants of the square primitive arrangement to $a=L+2H+2L_{lig}$and those of the centred rectangular arrangement to $a=2\left( L+H \right)+4L_{lig}$and $b=2L+4L_{lig}$, where $L_{lig}$ is the maximum stretched length of the ligand. We highlight that this defines the maximal spatial contribution of the ligands, assuming full surface coverage, complete extension, and no interdigitation with neighboring NCs. A maximum ligand extension length of *L_lig_*= 2.29 nm is considered for oleic acid, as reported in ^[3]^, and it is assumed that oleylamine has a similar full extension length. All computed 2D packing fractions for armed CsPbBr_3_ NCs of experimentally determined sizes are reported in **Table S4**.

**Table S4.** Calculated packing fractions for the experimental core-to-arm-size ratios without and with ligands consideration assembled into square and centred rectangular monolayers.

|  | *H, nm* | *L, nm* | *x* | $\eta$*_2D,sp_* | $\eta$*_2D,sp_(lig)* | $\eta$*_2D,cr_* | $\eta$*_2D,cr_(lig)* |
| --- | --- | --- | --- | --- | --- | --- | --- |
| Long arm | 8.9 | 22.9 | 0.39 | 0.81 | 0.65 | 0.92 | 0.67 |
| Middle arm | 5.7 | 19.8 | 0.29 | 0.87 | 0.66 | 0.84 | 0.58 |
| Short arm | 3.7 | 20.7 | 0.18 | 0.93 | 0.69 | 0.73 | 0.50 |

**Trilayers.** The 3D packing fraction (volume fraction of the unit cell occupied by the inorganic NC) is denoted as $\eta$*_3D,i_(x)* where is the arrangement of the CsPbBr_3_ NCs in the trilayer.

The first arrangement is described by a simple cubic (SC) lattice with space group *Pm-3m* (No. 221) containing one NC in Wyckoff position 1a. The lattice constant is $a=L+2H$, hence:

$$\eta_{3D,SC}= \frac{L^{3}+6L^{2}H}{{(L+2H)}^{3}}, \eta_{3D, SC}(x)= \frac{1+6x}{\left( 1+2x \right)^{3}}$$

The second arrangement is described by a body-centred cubic (BCC) lattice with space group *Im-3m* (No. 229) containing two NCs in Wyckoff positions 1a and 1b. The lattice constant is $a=2L$, hence:

$$\eta_{3D,BCC}= \frac{2(L^{3}+6L^{2}H)}{{8L}^{3}}, \eta_{3D, BCC}(x)= \frac{1+6x}{4}$$

Here again, the presence of ligands on the surface of the NCs can increase the lattice constants of both simple cubic and body centred cubic arrangements, up to $a=L+2H+2L_{lig}$ and $a=2L+4L_{lig}$respectively. This represents the maximal spatial contribution of the ligands, assuming full surface coverage, complete extension, and no interdigitation with neighboring NCs. A maximum ligand extension length of *L_lig_*= 2.29 nm is considered for oleic acid, as reported in ^[3]^, and it is assumed that oleylamine has a similar full extension length. All computed 3D packing fractions for armed CsPbBr_3_ NCs of experimentally determined sizes are reported in **Table S5**.

**Table S5.** Calculated packing fractions for the experimental core-to-arm-size ratios without and with ligands consideration assembled into simple cubic and body-centred cubic superlattices.

|  | *H, nm* | *L, nm* | *x* | $\eta$*_3D,SC_* | $\eta$*_3D,SC_(lig)* | $\eta$*_3D,BCC_* | $\eta$*_3D,BCC_(lig)* |
| --- | --- | --- | --- | --- | --- | --- | --- |
| Long arm | 8.9 | 22.9 | 0.39 | 0.60 | 0.43 | 0.83 | 0.48 |
| Middle arm | 5.7 | 19.8 | 0.29 | 0.70 | 0.46 | 0.68 | 0.37 |
| Short arm | 3.7 | 20.7 | 0.18 | 0.83 | 0.53 | 0.52 | 0.28 |
| Nanocubes | 0 | 10.0 | 0.00 | 1.00 | 0.32 | - | - |

**References**

[1] Snyder, L. R. Classification off the Solvent Properties of Common Liquids. *J Chromatogr Sci* 1978, *16* (6), 223–234. https://doi.org/10.1093/chromsci/16.6.223.

[2] Lee, K., Cho, Y., Kim, J.C. *et al.* Catalyst-free selective oxidation of C(sp^3^)-H bonds in toluene on water. *Nat Commun* 2024, 15, 6127. https://doi.org/10.1038/s41467-024-50352-7.

[3] Travesset, A. Soft Skyrmions, Spontaneous Valence and Selection Rules in Nanoparticle Superlattices. *ACS Nano* 2017, 11, 6, 5375–5382. https://doi.org/10.1021/acsnano.7b02219.
