# Supplementary figures and images for: Arm‐Length‐Controlled CsPbBr3 Nanocrystals for Tunable Optical and Assembly Behavior
Source: Adv Mater. 2026 Mar 24;38(23):e19211. doi: 10.1002/adma.202519211 (PMC13103628; doi:10.1002/adma.202519211)

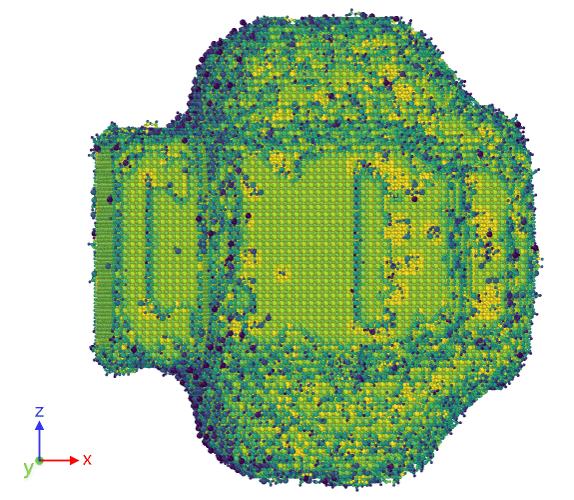

Supplement: Supplementary file 2 — Supporting File 2: adma72815‐sup‐0002‐MovieS1.gif. [file ADMA-38-e19211-s003.gif]

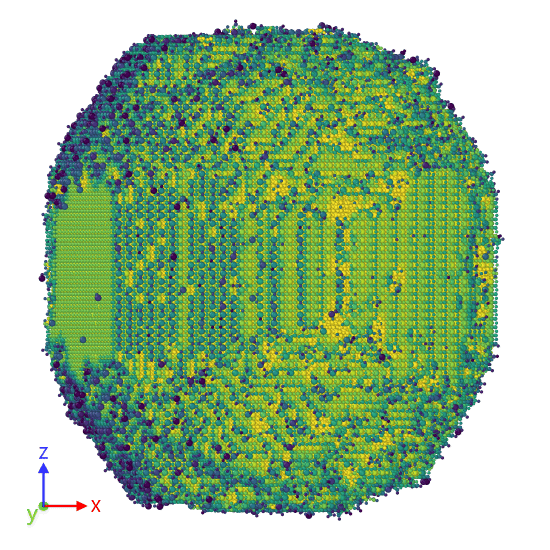

Supplement: Supplementary file 3 — Supporting File 3: adma72815‐sup‐0003‐MovieS2.gif. [file ADMA-38-e19211-s002.gif]
